# The effect of 3-month finasteride challenge on biomarkers for predicting cancer outcome on biopsy: Results of a randomized trial

**DOI:** 10.1371/journal.pone.0204823

**Published:** 2018-10-09

**Authors:** Javier Hernandez, Jonathan Gelfond, Martin Goros, Michael A. Liss, Yuanyuan Liang, Donna Ankerst, Ian M. Thompson, Robin J. Leach

**Affiliations:** 1 Department of Urology, The University of Texas Health San Antonio, San Antonio, Texas, United States of America; 2 Department of Epidemiology and Biostatistics, The University of Texas Health San Antonio, San Antonio, Texas, United States of America; 3 Department of Epidemiology & Public Health, University of Maryland School of Public Health, Baltimore, Maryland, United States of America; 4 Life Sciences Mathematics Unit, Technical University Munich, Munich, Germany; 5 CHRISTUS Santa Rosa Hospital–Medical Center, San Antonio, Texas, United States of America; 6 Department of Cell Systems and Anatomy, The University of Texas Health San Antonio, San Antonio, Texas, United States of America; University of Sydney, AUSTRALIA

## Abstract

**Background:**

Finasteride, a 5-alpha reductase inhibitor may have effects on biomarkers such as prostate-specific antigen (PSA) that could be leveraged to improve screening.

**Objective:**

To determine the predictive characteristics of biomarkers for prostate cancer for cancer on biopsy following 3 months of finasteride use compared with placebo.

**Design, setting and participants:**

383 men from multiple clinical sites with intermediate prostate cancer risk, without history of prostate cancer, were randomly allocated in a double-blinded manner, 4:1, to receive either finasteride or placebo for 90 days at which time a prostate biopsy was performed.

**Outcome measurements and statistical analysis:**

The primary outcomes were associations of biomarkers with prostate cancer that were tested using multiple logistic regression and area under the receiver operating curves (AUC). Biomarkers for PCA risk (PCA3, TMPRSS2:ERG (T2:ERG) gene product, and PSA) were measured at baseline and at biopsy in a blinded fashion to assess the predictive performance of baseline levels, 90-day levels, and measures of change relative to standard predictors.

**Results and limitations:**

A total of 292 (233 finasteride; 59 placebo) randomized patients underwent biopsy and were analyzed. On finasteride, baseline and 90-day measures of PCA3 and T2:ERG had similar moderate discrimination capacity with AUCs 62 to 65% (p-values < 0.001 and 0.001, respectively), but their rates of change had no discrimination ability (AUC 51%, (95% CI 43 to 60% p = 0.72) and 48% (95% CI 44 to 60%, p = 0.62), respectively).) Relative to baseline, the 90-day PCA3 and PSA decreased in the finasteride group by 25% and 50%, respectively (both p<0.001). T2:ERG had a smaller, non-significant change post finasteride treatment (p = 0.08).

**Conclusions:**

Short-term finasteride therapy did not improve performance of the most commonly-employed prostate cancer biomarkers. Threshold values for new biomarkers of prostate cancer should be interpreted with caution in patients receiving finasteride until formal validation of test performance in these patients is conducted.

**Patient summary:**

Three months of finasteride treatment did not increase the accuracy for predicting the outcome on prostate biopsy but did have a significant effect on biomarker values. Adjustments to thresholds for biopsy for men on finasteride are proposed.

**Trial registration:**

ClinicalTrials.gov, NCT01296672.

## Introduction

Prostate cancer (PCA) is the most common non-cutaneous cancer in men living in the United States. A major focus for disease control has been early diagnosis using the prostate-specific antigen (PSA) blood test. There are many challenges with PSA testing. Many patients with prostate cancer, including aggressive cancers, have normal PSA levels[1]. As a screening biomarker, PSA alone has a high false positive rate [2]. Furthermore, the majority of prostate biopsies prompted by elevated PSA tests find no cancer, and these biopsies incur pain, risks of fever, prostatitis, and urine retention [3, 4] as well as substantial costs to the healthcare system [5]. The limitations of PSA screening led the US Preventative Services Task Force to initially recommend against the use of PSA for screening in 2012 [6], although recent changes recommend offering PSA testing to at-risk men age 55–69. Nonetheless, improvements in diagnosis are necessary, and there are promising biomarkers that have evidence for improving screening accuracy. Included among these are the noncoding RNA known as Prostate cancer antigen 3 (PCA3) [7] and TMPRSS2:ERG (T2:ERG) fusion gene. ERG is a member of the Erythroblast Transformation-Specific (ETS) gene family and is commonly fused in prostate cancer to the TMPRSS2 gene (Transmembrane Protease, Serine 2)[8]. These markers are measured by quantitating the level of their RNA transcripts in post-DRE (digital rectal examine) urine from subjects normalized against PSA transcripts.

An intriguing observation related to biomarker performance arose from the Prostate Cancer Prevention Trial (PCPT) in which 18,882 men were randomized to finasteride or placebo with all cancer-free men after 7 years of study recommended to undergo an end-of-study biopsy. The performance of PSA for prostate cancer detection as well as for detection of high-grade disease was significantly greater in those randomized to finasteride [9]. Several additional studies have suggested that a smaller reduction in PSA after initiating a 5-alpha reductase inhibitor is more likely associated with prostate cancer [10–14]. The study’s primary objective was to determine if a brief, 3-month challenge with finasteride would improve selection of patients with elevated PSA for prostate biopsy as well as to examine the impact of finasteride on other prostate cancer biomarkers. The selection of this interval was based on several factors. Finasteride will decrease PSA levels roughly by 50% within 6 months. However, most of the PSA decrease occurs within the first three months. Additionally, we did not want to risk the possibility of delaying a high grade prostate cancer diagnosis for 6 months. The primary hypothesis was that the change in PSA after 3-month finasteride treatment would discriminate between men with and without prostate cancer.

## Materials and methods

### Study design

The eligibility criteria specified men 55 years or older with or without prior negative prostate biopsy who had an estimated prostate cancer risk of between 20 and 60% based upon the prostate cancer risk calculator (PCPT) version 1.0 and were willing to undergo biopsy at 3-months. The exclusion criteria included any prior history of prostate cancer and men with whom there was concern of disease progression prior to three months. Men were recruited for the trial between 2011 and 2016. The institutional review board approved the study at three sites (Medical Arts and Research Center [MARC], University Health System (Bexar County Hospital), and the Audie L. Murphy Memorial Veterans Affairs [VA] Hospital San Antonio, Texas in San Antonio, TX).

After providing written informed consent, eligible subjects were sequentially allocated to receive a bottle containing 100 tablets of finasteride 5 mg or placebo, respectively, and were instructed to take one tablet daily for 3 months. A computer-generated randomized list was created and held by Merck Sharp & Dohme Corp and not disclosed to the study personnel, the treating physician, and the patients who were blinded to treatment allocation throughout the duration of the study. This list was randomized in a 4:1 (finasteride:placebo) ratio and the study pharmacist allocated patients sequentially to receive bottles with identical appearing pills according to the randomization list. The 4:1 ratio was utilized since the primary endpoint of the study was to compare the changes in PSA as a predictor of cancer for men who were taking finasteride.

**Fig 1:**
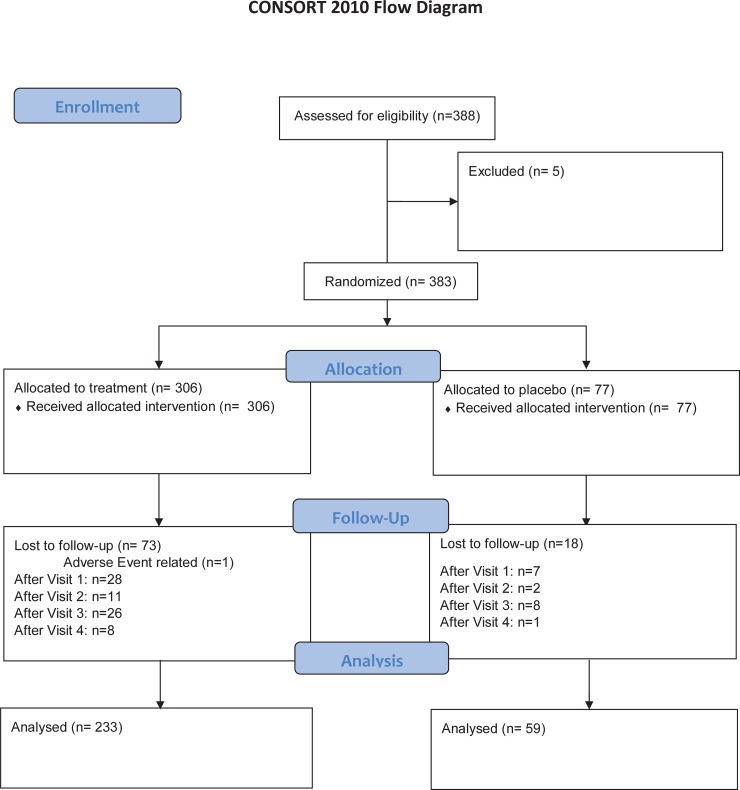
CONSORT patient flow diagram for the finasteride challenge trial. Patients were excluded based on their risk of high grade disease based updated clinical data. PSA measurements were often repeated to be within the 6 month window at time of enrollment. The loss to follow-up subjects included men who withdrew from the study, many of whom decided to postpone their prostate biopsies.

Baseline assessments included age, family history of prostate cancer, and ethnicity. PSA was measured monthly starting at randomization (baseline, 30, 60, and 90 days) by Quest Diagnostics. At baseline and at 3-months (90 days ± 7 days), subjects underwent evaluation for PSA, digital rectal examination (DRE), PCA3, the most common ETS gene fusion TMPRSS2:ERG (T2:ERG). PSA assessments were performed prior to DREs. Post-DRE urine samples were evaluated by Hologic, Inc. for levels of PCA3 and T2:ERG. At 90 ± 14 days, subjects underwent 12-core prostate biopsy. Each core was fixed in formalin and processed for pathologic assessment and individually read for the presence of prostate cancer (Gleason grade, cancer percent per core, perineural invasion), presence of prostatic intraepithelial neoplasia, atypia, or inflammation. After biopsy, subjects were followed clinically using local standard of care. The patient flow diagram is shown in Fig 1.

### Statistical analyses

The primary outcome was the area-underneath-the-receiver-operating-characteristic curve (AUC) associating change in PSA after 3-months of finasteride treatment and the risk of diagnosis of prostate cancer at 3-month biopsy. The secondary outcomes were finasteride related changes in the biomarkers over the same period. Change over time in biomarkers between the cancer and no cancer groups were compared using the Wilcoxon test. Specifically, the rates of change were calculated as the logarithm of the ratio of the 3-month to baseline value. The AUC for association with prostate cancer was calculated using the Wilcoxon statistic for the baseline level and 3-month change in biomarkers [8]. Adjustment factors for biomarkers under finasteride were computed as the 100/(100-D) where D is the percent decrease at 3-months relative to baseline for the finasteride arm. Multivariable logistic regression was used to assess the independent predictive value of log_e_ transformed PCA3 and T2:ERG to the standard prostate biopsy risk factors, including PSA, DRE, African American race, age, history of a negative biopsy and prostate cancer family history.

Sample size was selected to ensure sufficient accuracy for characterizing sensitivity and specificity of a 3-month finasteride PSA change, assuming a 40% positive biopsy rate in study participants. Finasteride was assumed to reduce PSA in men with and without cancer by 10–15% and 34%, respectively, corresponding to an additive change in the logarithmic PSA scale that was used to approximately a Gaussian distribution. In men with cancer, this corresponds to a decrease of–log(1–0.15) = 0.163 to–log (1–0.10) = 0.105, and in men without cancer, this corresponds to a decrease of -log(1.034) = 0.416. Under these assumptions, a sample size of 360 (144 cancer cases and 216 controls) in finasteride arm achieves a 75%– 90% power to test null hypothesis that there is no difference between cases and controls in 3-month finasteride PSA change. All statistical tests were performed at the two-sided 0.05 significance level and all analyses were performed in the R statistical package. The accrual rate was much longer than expected because of the changes in the PSA recommendation by the US Preventative Services Task force. After extending the accrual period and with continued low enrollment, analysis was perform by a biostatistician (JG) prior to attaining the target sample size, and accrual was stopped based on these findings.

## Results

### Patient characteristics and cancer outcomes

After the planned accrual period of four years, the enrollment was extended by one year to lessen the effects of slow accrual on study power. A total of 383 men were randomized (306 on finasteride, 77 on placebo); baseline characteristics were similar between the two arms. A total of 233 patients receiving finasteride and 59 receiving placebo completed the study and underwent a prostate biopsy (Table 1); these subjects were utilized in the analysis (Fig 1). Approximately half of the patients randomized to each arm were diagnosed with prostate cancer and approximately half of all cancers on each arm were Gleason grade ≥7 (Table 1). Adverse events for all randomized patients are summarized in S1 Table. The most frequent adverse events in the finasteride arm were related to sexual function (decreased libido 1% (3/306); impotence 0.7% (2/306); painful ejaculation, enlarged vase deferens, and epididymitis were reported in <1% each (1/306)). Cardiovascular adverse events (Chest pain, arrhythmia, syncope, stent placement) were rare in both arms (<1%, 1/306 each), and not significantly different in the two arms (p>0.2).

**Table 1:** Baseline patient characteristics and cancer outcomes for the 292 patients in analysis set.

	Finasteride (n = 233)	Placebo (n = 59)	P-Value
Mean pt age (SD)	64.62 (4.95)	65.37 (4.25)	0.286
No. pt age (%):			0.265
< 60	45 (19.3)	6 (10.2)	
60–69	152 (65.2)	46 (78.0)	
70–79	35 (15.0)	7 (11.9)	
≥ 80	1 (.4)	0 (0.0)	
Mean PSA (SD)	5.7 (2.0)	5.6 (1.9)	0.599
No. Race (%):			0.876
Non-Hispanic white	110 (47.2)	29 (49.2)	
Non-Hispanic black	30 (12.9)	6 (10.2)	
Hispanic	89 (38.2)	24 (40.7)	
Other	4 (1.7)	0 (0.0)	
No. Prior biopsy (%):			0.325
Yes	35 (15.0)	12 (20.3)	
No	198 (85.0)	47 (79.7)	
No. DRE (%):			0.406
Abnormal	16 (6.9)	6 (10.2)	
Normal	216 (92.7)	52 (88.1)	
Missing	1 (0.4)	1 (1.7)	
No. family history (%):			0.847
Yes	41 (17.6)	9 (15.3)	
No	192 (82.4)	50 (84.7)	
No. cancer status (%):			0.772
Yes	116 (49.8)	28 (47.5)	
No	117 (50.2)	31 (52.5)	
No. Gleason score (%):			0.797
6	56 (24.0)	13 (22.0)	
7	48 (20.6)	14 (23.7)	
8	8 (3.4)	1 (1.7)	
9	1 (0.4)	0 (0.0)	
Missing	3 (2.6)	0 (0.0)	

### Markers and association with cancer

In the finasteride group, PSA at baseline had a slight discriminative ability (AUC 58%, p = 0.04), but the 90-day PSA did not predict cancer status (AUC 53%, p = 0.43). This was primarily due to the inclusion criterion of moderate risk on the PCPT Risk Calculator (version 1), which essentially amounts to a high PSA for all participants, as can be seen by the distribution of PSA in Table 2.

**Table 2:** Biomarker characteristics at baseline, 90-day final measurement and for the ratio of final to baseline measurement. AUC is area under the receiver operating characteristic curve. The first (Q1) and third (Q3) quartiles for biomarkers are given as [Q1,Q3]. The 95% confidence intervals are given for AUC estimates as [Lower limit, Upper limit].

Biomarker	Time	No Cancer	Finasteride Cancer	AUC	No Cancer	Placebo Cancer	AUC
PCA3 Score	Baseline	23.4	39.65	0.65	12.89	65.79	0.84
		[11.27,40.39]	[18.86,81.49]*#	[0.58, 0.72]^	[9.8,34.5]	[44.6,82.21]*	[0.73, 0.96]^
	Final	17.26	31.24	0.63	16.99	65.52	0.8
		[8.31,33.21]	[13.96,58.09]*#	[0.56, 0.71]^	[9.05,35.77]	[30.67,83.58]*	[0.68, 0.92]^
	Ratio	0.74	0.75	0.51	1.01	0.96	0.45
		[0.5,1.08]	[0.53,1.1]#	[0.43, 0.6]	[0.73,1.3]	[0.75,1.1]	[0.39, 0.7]
T2:ERG Score	Baseline	2.64	6.82	0.62	1.81	10.07	0.62
		[0.09,14.16]	[0.15,59.8]*	[0.55, 0.69]^	[0.09,20.95]	[2.26,98.44]	[0.47, 0.76]
	Final	1.68	8.77	0.63	8.34	26.09	0.68
		[0.09,11.67]	[0.23,34.6]*	[0.55, 0.7]^	[0.09,25.26]	[3.23,134.52]*	[0.54, 0.82]^
	Ratio	1	0.67	0.48	1	1.72	0.56
		[0.3,2.36]	[0.23,2.57]	[0.44, 0.6]	[0.41,3.68]	[0.66,6.1]	[0.41, 0.71]
PSA	Baseline	5.03	5.43	0.58	5.1	5.15	0.54
		[4.2,6.2]	[4.48,6.82]*	[0.5, 0.65]^	[4.17,6.17]	[4.65,7.21]	[0.39, 0.69]
	Final	2.6	2.7	0.53	4.1	4.9	0.61
		[2.1,3.5]	[2.1,3.7]#	[0.45, 0.61]	[3.3,5.65]	[3.7,6.55]	[0.47, 0.76]
	Ratio	0.53	0.5	0.47	0.82	0.87	0.61
		[0.41,0.63]	[0.4,0.62]#	[0.45, 0.6]	[0.63,0.95]	[0.79,1]	[0.46, 0.76]

* P-value between cancer and no cancer less than .05.

# P-value between finasteride and placebo less than .05.

^ P-value less than .05.

In the finasteride arm, the 90-day change in PCA3 and T2:ERG did not discriminate men with and without cancer (AUC 51% (95% CI 43 to 60%, p = 0.72) and 48% (95% CI 44 to 60%), respectively,) however, baseline and 90-day measures of PCA3 and T2:ERG were moderately predictive of cancer with AUCs ranging from 62 to 65% (p-values < 0.002 to <-0.001, Table 2) In the placebo arm, the baseline and 90-day AUCs of PCA3 were higher than the finasteride arm (84% and 80%, respectively, comparison to the finasteride arm, 65% and 63%, respectively). As in the finasteride arm, the 90-day change in PCA3 and T2:ERG did not predict cancer status (AUC ~50%, p = 0.55 and p = 0.46, respectively). The AUCs of baseline T2:ERG (62%) and 90-day T2:ERG (63%) were no better on finasteride than on placebo (both p-values > 0.05), and in the placebo arm, only the 90-day T2:ERG AUC was significantly different than 50% (p = 0.02).

Baseline and 90-day measures of PCA3 and T2:ERG all contributed independent information for prostate cancer detection on finasteride (all p-values < 0.01), but rates of change did not (Table 3).

**Table 3:** Independent predictive value of PCA3 and T2ERG to the standard risk factors for detection of prostate cancer on biopsy on finasteride: PSA, DRE, age, African American race, negative prior biopsy and family history. Odds ratios are relative to unit changes in the log_e_ of the biomarkers.

Time of Measure	Biomarker	Odds Ratio	95% Confidence Interval	P-Value
Baseline	PCA3 Score	1.12	[1.05, 1.2]	<0.001
	T2 Score	1.04	[1.02, 1.07]	<0.001
Final	PCA3 Score	1.09	[1.02, 1.17]	0.008
	T2 Score	1.05	[1.02, 1.07]	<0.001
Ratio	PCA3 Score	1.01	[0.91, 1.11]	0.895
	T2 Score	1.00	[0.97, 1.03]	0.841

### Proposed inflation factors for use of biomarkers for patients on finasteride

**Fig 2:**
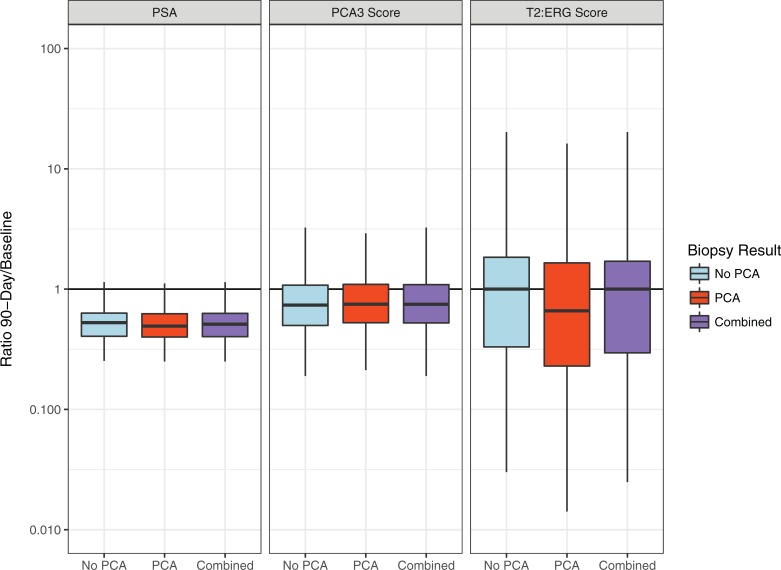
PCA3, T2:ERG and PSA ratios on finasteride for determination of inflation factors; PCA = prostate cancer. Boxplots depict the 25th, 50th, and 75th percentiles. Outliers are not shown due to extreme variability of T2:ERG ratios.

In the finasteride arm, PCA3 and PSA decreased at 90-days relative to baseline while T2:ERG did not change. See Fig 2. For PCA3, the median, first and third quartiles [Q1, Q3] of the percent decrease for men with PCa, men without PCa, and all men were -25 [-47, +9]%, -26 [-50, +8] %, and -25 [-48, +9] % (all p-values<0.001), respectively. The median, [Q1, Q3] percent change for T2:ERG in men with PCa (-33 [-77, +166]%) and without PCa (0 [-70, +136, ]%) were not significantly different from 0 (p>0.05). For PSA, the median, [Q1, Q3] percent changes in men with PCa (-51%, [–60, –38 ]%), without PCa (-47%, [–59, –37 ] %) and all men (49%, [–60, –37] %) indicate statistically significant decreases (p<0.0001). These results suggest that finasteride substantially reduces PCA3 and PSA, potentially causing underestimated risk calculations using these biomarkers. Based on changes in men with and without cancer in the finasteride arm, these results suggest that an inflation factor of 1.33 = 100/(100–25%) for PCA3, (i.e., inflating PCA3 values by 33%) would make them comparable for recommended thresholds; as expected, an inflation factor of 2.0 = 100/(100–49%) for PSA would be appropriate.

## Discussion

The management of an asymptomatic man with an elevated PSA is a clinical challenge. While the PSA test is highly-associated with risk of prostate cancer and the risk of potentially-lethal, high-grade prostate cancer, many men undergoing prostate biopsy will be found to be cancer-free or to harbor low-grade, low-volume disease. Higher PSA levels in these men are often due to larger prostate volumes; as these men often have concomitant urinary symptoms, it has been a common practice to treat them with antibiotics or a 5-alpha reductase inhibitor and monitor the fall in PSA. The use of 5-alpha reductase inhibitors to reduce PSA, a greater fall in PSA is often ascribed to prostatic hyperplasia. Traditionally, the prescriber would expect a 50% reduction in finasteride and if the PSA did not fall to ½ the original PSA then cancer may be cause of persistent PSA elevation. Additionally, men with higher PSA values who have had a prior negative biopsy and who are then treated with finasteride may subsequently undergo testing with secondary screening tests such as PCA3. This study was designed to address both the kinetics of change in biomarkers during a 3-month challenge with finasteride as well as biomarker performance and cut-points before and after finasteride administration.

We found that changes in biomarkers after a brief exposure to finasteride did not improve prostate cancer detection. While data from PCPT clearly show improved PSA test performance in men on long-term finasteride therapy, shorter-term administration in this study did not accrue this benefit. The strength of this study included a concurrent assessment of test performance of other biomarkers before and after finasteride administration. It is clear that different thresholds for recommending biopsy should be used during finasteride therapy. In the setting of short-term administration*¸* our preliminary data suggest that patient with a threshold value of PCA3, for example, should be decreased from 25 to approximately 19. Several studies have documented the specificity of PCA3, but clinical trials have generally excluded men taking medications such as finasteride that could affect PSA [15, 16]. It is also a concern that several published studies have not included medication data nor reported if patients were taking this class of medication [17,18,19]. The implications of finasteride altering biomarker test performance could also impact men on active surveillance who are receiving a five-alpha reductase inhibitor [20, 21]. We suspect that, like PSA and PCA3, other prostate cancer biomarkers may be significantly altered by finasteride and should be interpreted cautiously in patients receiving this medication class.

A key limitation of the study is that the target sample size was not attained (n = 306 analyzed vs. target of n = 360), which resulted in lower statistical power. However, the primary endpoints were the AUCs of the changes in PCA3 and T2:ERG biomarkers, and these endpoints were estimated with sufficient accuracy to conclude that the ability of biomarker change to predict cancer status was poor. The study was also limited by a higher than anticipated dropout rate (~35%), but the main reason for dropout was unwillingness to undergo biopsy for reasons that are unrelated to biomarker changes, and thus, are not likely to bias these conclusions.

In conclusion, PSA changes with short-term administration of finasteride should not be used to determine the need for a prostate biopsy in a man suspected based on PSA to harbor prostate cancer. Other biomarkers should be interpreted with caution in men receiving finasteride.

## Supporting information

S1 Checklist CONSORT 2010 Checklist. (DOC)

S1 Table Adverse events stratified by treatment. A patient may have had a specific event more than once.
(DOCX)

S1 File Finasteride challenge protocol developed in 2010 utilized in current study. (DOC)

S1 Analytical Set Developed from the finasteride challenge study. (XLS)
