# Supplementary material for: The effect of 3-month finasteride challenge on biomarkers for predicting cancer outcome on biopsy: Results of a randomized trial
Source: PLoS One. 2018 Oct 9;13(10):e0204823. doi: 10.1371/journal.pone.0204823 (PMC6177134; doi:10.1371/journal.pone.0204823)
Supplement: S1 Table — A patient may have had a specific event more than once. (DOCX) [file pone.0204823.s002.docx]

| **Event** | **Adverse Event** | **Finasteride (n=306)** | **Placebo (n=77)** | **P-value** |
| --- | --- | --- | --- | --- |
| Adverse events No. (%) |  | 42 (14) | 7 (9.1) | 0.34 |
| Drug-related event No. (%) |  | 10 (3.3) | 1 (1.3) | 0.70 |
| Event leading to study withdrawal No. (%) |  | 1 (0.3) | 0 (0.0) | 1.00 |
| Adverse events related to sexual function | Decreased libido | 3 (1.0) | 0 | 1.00 |
| No. (%) | Impotence | 2 (0.7) | 0 | 1.00 |
|  | Painful ejaculation | 1 (0.3) | 0 | 1.00 |
|  | Enlarged vas deferens | 1 (0.3) | 0 | 1.00 |
|  | Epididymitis | 1 (0.3) | 0 | 1.00 |
| Cardiovascular | Chest pain | 1 (0.3) | 0 | 1.00 |
| No. (%) | Syncope | 0 | 1 (1.3) | 0.20 |
|  | Arrhythmia | 1 (0.3) | 0 | 1.00 |
|  | Stent placement | 0 | 1 (1.3) | 0.20 |
|  | Tachycardia | 1 (0.3) | 0 | 1.00 |
