# Supplementary material for: The effect of 3-month finasteride challenge on biomarkers for predicting cancer outcome on biopsy: Results of a randomized trial
Source: PLoS One. 2018 Oct 9;13(10):e0204823. doi: 10.1371/journal.pone.0204823 (PMC6177134; doi:10.1371/journal.pone.0204823)
Supplement: S1 File — (DOC) [file pone.0204823.s003.DOC]

**Improving Prostate Biopsy Efficiency:  The Finasteride Challenge Test**

# Protocol

# Version 12

# Dated 01/03/2014

**Study Sites:** **The University of Texas Health Science Center at San Antonio**

**(UTHSCSA)**

**Medical Arts and Research Center (MARC) at UTHSCSA**

**University Hospital Downtown, Robert B. Green campus**

**South Texas Veterans Health Care System (STVHCS), Audie L. Murphy Division**

**Funding: National Institutes of Health (NIH)**

**Table of Contents**

Responsible Entities and Staff……………………………………… 3

1. Background and Significance….………………………………….. 4-6
2. Hypothesis/Specific Aims……………….…………………………7
3. Research Design and Methods……………………………………. 7

3.1 Primary Objective………………………………………………7

3.2 Secondary Objective……………………………………………8

- 1. 3.3 Research Procedures……………………………………………10
  2. 3.4 Clinical Procedures……………………………………………..10
  3. 3.5 Subject Population………………………………………………10
  4. 3.6 Recruitment and consent procedures…………………………....11

4. Human Subjects……………………………………………………12

4.1 Compensation…………………………………………………. 12

4.2 Risks to subject……………………………………………….. 12

4.3 Special precautions……………………………………………. 13

4.4 Alternative treatments………………………………………… 13

4.5 Confidentiality………………………………………………… 13

5. Safety Monitoring…………………………………………………. 14-16

6. Data Analysis…………………………………………………..… 15-23

7. Literature Cited…………………………………………………… 24-29

**APPENDIX 1 – Drug Formulation**

**APPENDIX 2 –CASE REPORT FORMS**

**Responsible Entities and Staff**

Principal Investigator: Javier Hernandez, M.D.

Assistant Professor

Department of Urology

The University of Texas Health Science Center San Antonio

Sub-Investigators: Ian Thompson, Jr.,M.D.

Professor

Department of Urology

The University of Texas Health Science Center San Antonio

Joseph W. Basler, M.D.

Professor

Department of Urology

The University of Texas Health Science Center,

San Antonio

Stephen Kraus, M.D.

Professor

Department of Urology

The University of Texas Health Science Center,

San Antonio

Jonathan Gelfond, M.D., Ph.D.

Assistant Professor

Department of Epidemiology and Biostatistics

The University of Texas Health Science Center,

San Antonio

Marlo Nicolas, M.D.

Assistant Professor

Department of Pathology

The University of Texas Health Science Center,

San Antonio

Thomas Rozanski, MD

Professor

Department of Urology

The University of Texas Health Science Center,

San Antonio

Robert Svatek, M.D.

Assistant Professor

Department of Urology

The University of Texas Health Science Center,

San Antonio

Timothy Tseng, M.D.

Assistant Professor

Department of Urology

The University of Texas Health Science Center,

San Antonio

Robin Leach, PhD

Professor

Departments of Structural and Cellular Biology and Urology

The University of Texas Health Science Center,

San Antonio

**1. Background**

Prostate cancer is the most common non-cutaneous cancer in U.S. men. A major focus for disease control is early diagnosis using the prostate specific antigen (PSA) blood test and prostate digital rectal examination (DRE); these tests are used by over half of U.S. men annually. The challenge with PSA testing is that most men with a higher PSA do not have prostate cancer and, many cancers including aggressive cancers have normal PSA levels. Because of this, about 75% of prostate biopsies prompted by an elevated PSA test finds no cancer. It is clear that an improved method of diagnosis is required.

Rationale for this proposed project: Our observations including the relationship between change in PSA and risk of prostate cancer from the Prostate Cancer Prevention Trial (PCPT) provide an opportunity for a simple method to improve current prostate cancer screening by reducing the number of unnecessary (negative) prostate biopsies. We propose to determine if a 3-month finasteride ‘challenge’ can improve the performance of prostate cancer screening.

**Preliminary Studies from the Prostate Cancer Prevention Trial (PCPT)**

Our group of investigators has been responsible for some of the most important advances in prostate cancer. Dr. Thompson served as the Clinical Coordinator of the 18,882-subject PCPT. In this study, 9459 men were randomized to placebo; based on these men, we developed the first multiple risk factor, on-line prostate cancer risk calculator (<http://deb.uthscsa.edu/URORiskCalc/Pages/uroriskcalc.jsp>). The PCPT risk calculator has become an essential tool for physicians around the world managing patients who are at risk of prostate cancer and has provided a number of important discoveries into how PSA should be used as a screening test. We recently pursued the implications of a study by Guess in our larger population. He found that PSA in a man receiving finasteride functions better as a test for prostate cancer.^[1]^ We examined men in the PCPT who underwent prostate biopsy; 5112 men in the placebo group and 4579 men in the finasteride group were studied. We found that the area under the ROC curve (AUC) for PSA was significantly higher for prostate cancer detection and for detection of high grade (Gleason > 7) disease among men receiving finasteride. ^[1]^ Maintaining constant specificity, we found highly significant increases in sensitivity for cancer detection. For example, using a cutoff of 4.1 ng/mL, PSA had a 24% sensitivity for prostate cancer detection; in men receiving finasteride, using a cutoff with the same specificity, sensitivity for cancer detection was increased to 37.8%. This analysis led to the recent update of the calculator by confirming that doubling of the PSA in a man on finasteride provides additive predictive value for the assessment of risk of prostate cancer. ^[2]^

PCPT data are based on long-term exposure to finasteride; in these men, biopsy was performed 5-7 years after starting study drug. For a man who is considering biopsy *at the present time*, waiting such a period to evaluate change in PSA is unacceptable; if a high-grade tumor were present, it would miss the opportunity for cure. Data from previous studies of finasteride suggest that most of the PSA fall occurs in the first few months after starting the drug with most of the fall within 6 months.^[3]^ Clinically, a 6-month wait to evaluate PSA response may also not be acceptable. Clinical data show that a 3-month wait before treatment has no impact on prostate cancer outcomes;^[4,5]^ other data show that after three months of treatment with finasteride, about 60% of the total fall in PSA will occur.^[6]^ We have conducted a preliminary analysis in the PCPT. Of 958 men with an initial PSA < 1.8 ng/mL and using men with a 65% or greater fall in PSA set as the reference group, there was almost a 3-fold greater risk of prostate cancer in the group of individuals whose PSA did not change or increased during the period of finasteride treatment. The risk of prostate cancer was inversely associated in a stepwise fashion with the degree of fall in PSA.

Further increasing the compelling nature of this proposal are two recent studies from the PCPT that suggest that men who are undergoing PSA testing should be offered finasteride as a chemoprevention agent for prevention of prostate cancer. These two studies address the two final questions about the utility of finasteride for prostate cancer prevention. Previous publications have demonstrated the following:

1. Finasteride reduces a man’s risk of prostate cancer by approximately 25%.^[7]^
2. Finasteride improves the performance (including sensitivity) of serum PSA for cancer screening.^[1]^
3. Finasteride improves the performance of digital rectal examination for cancer screening.^[8]^
4. Finasteride increases the sensitivity of prostate biopsy for the detection of prostate cancer and for the detection of high grade prostate cancer.^[9]^
5. Side effects with finasteride are minimal.^[10]^
6. Finasteride reduces the risk of high grade prostatic intraepithelial neoplasia, a premalignant lesion.^[11]^

Recent studies have analyzed the impact of finasteride’s two effects (reducing the risk of prostate cancer while concurrently helping the physician to detect the disease). The consensus of these studies is that finasteride reduces the risk of prostate cancer by more than 30% and also decreases the risk of aggressive disease while assisting in cancer detection.^[12]^ Concurrently, Lucia showed that tumors detected among men receiving finasteride were less extensive than those in men receiving placebo.^[13]^ We expect that with publication of these results and the release of the ASCO and AUA guidelines recommending offering finasteride to all men undergoing PSA testing, a substantial number of men will consider chemoprevention with finasteride^[14]^. If we prove that the change in PSA after 3 months of finasteride treatment improve prostate cancer detection, we expect that not only would the drug be used for chemoprevention of prostate cancer but also considered as front-line treatment for the man with an elevated PSA to determine if he truly will benefit from a prostate biopsy. The number of men affected by these findings exceeds one million annually in the U.S.

**Rationale for this application**. These observations provide an opportunity for a simple method to improve the performance of prostate cancer screening including the opportunity to significantly reduce the number of unnecessary (negative) prostate biopsies. We propose to determine if a 3-month finasteride ‘challenge’ can significantly improve the performance of screening for prostate cancer.

**SABOR (San Antonio center of Biomarkers Of Risk of prostate cancer)**

Our group has extensive experience with discovery and validation of prostate cancer biomarkers as part of our own ongoing study of men (SABOR cohort) with a design similar to the PCPT: annual evaluations including PSA and DRE with prostate biopsy determined based on clinical findings. This cohort has provided a platform for preliminary studies investigating PSA under short-term finasteride use and methodologies for incorporating new markers into the PCPT Risk Calculator. Many men from the SABOR cohort had responses to finasteride (initiated concurrent to their participation due to the development of BPH symptoms) supporting this study’s hypothesis. These men had increasing or higher PSA values due to concurrent BPH and enlarged prostates. They often then had one or more prostate biopsies, which were negative and were then placed on finasteride by their physician. In these men with negative biopsies, the PSA frequently fell dramatically. We hypothesize that finasteride given prior to biopsy could avoid prostate biopsies in many men in the future.

Our access to an ethnically diverse population and a record of inclusion of minorities is important as our findings will have a greater validity in the general U.S. population. In addition to a 'PSA Challenge Test' after finasteride, this study will evaluate new biomarkers of prostate cancer that may further improve on the ability of the clinician to segregate men with prostate cancer from those without prostate cancer that simply have a higher PSA value. The most promising markers at this time include: prostate cancer gene 3 (PCA3) and gene fusions (TMPRSS2:ERG) particular the TMPRSS2:ERG fusion. Including these non-drug challenge diagnostic tests will improve the scope of this application; positive results will translate into improved clinical outcomes including a reduction of unnecessary biopsies and their associated costs.

**2. Hypothesis and Specific Aims**

Our hypothesis is that the rate and magnitude of fall of PSA with a three-month exposure of finasteride will be greater in men without prostate cancer than in men with prostate cancer. The genesis of this hypothesis was three previous observations. (1) Most men with higher levels of serum PSA do not have prostate cancer; in these men a false-positive result is due to PSA production from hyperplastic epithelium Benign Prostatic Hyperplasia (BPH), a condition common in older men. (2) A collection of observations have been made that the degree of fall of PSA is greater in men without prostate cancer than in men with prostate cancer. (3) We have previously observed in the PCPT that in men receiving finasteride, serum PSA is a more sensitive and specific test for prostate cancer.

We propose a novel method to improve prostate cancer screening with PSA, using a 3-month treatment with finasteride, a drug used to treat BPH and proven to reduce a man’s risk of developing prostate cancer. We will also examine three additional promising tests that may further improve diagnosis of prostate cancer. The primary goal of this proposal is to determine if a 5-alpha reductase ‘challenge’ improves screening performance of PSA and DRE in men who are scheduled for prostate biopsy. Currently a higher PSA level leads to a recommendation for prostate biopsy, causing hundreds of thousands of unnecessary biopsies annually in the U.S. We will show that a three-month treatment with finasteride for men with high PSA levels will better predict the man who should have a prostate biopsy. PSA performance after finasteride ‘challenge’ will also be compared with new tests for prostate cancer. To this end, we propose the following specific aims:

• **Specific Aim 1:** Recruit a population of men at intermediate risk of prostate cancer (20%–60% risk) to a prospective, randomized, placebo-controlled clinical trial to receive either finasteride 5mg daily for 3 months or placebo before prostate biopsy.

• **Specific Aim 2:** Evaluate PSA velocity after a 3-month finasteride challenge as a marker for prostate cancer.

• **Specific Aim 3:** Characterize the operating characteristics of PSA and DRE at baseline and 3 months in finasteride and placebo groups and compare to the 3-month finasteride challenge as markers for disease.

• **Specific Aim 4:** Assess the independent diagnostic value of 3-month finasteride serum PSA velocity to the Prostate Cancer Prevention Trial (PCPT) prostate cancer risk calculator.

• **Specific Aim 5:** Assess the independent predictive value of new prostate cancer markers including PCA3, and a ETS gene fusion (TMPRSS2:ERG) on the performance of the PSA-based markers and, in combination or in place of the results of the finasteride ‘challenge’ test.

**3. Research Design and Methods**

**3.1 Primary Objective:** To determine if a five alpha reductase ‘challenge’ with finasteride improves the performance of the combination of PSA and DRE in men who are scheduled for prostate biopsy.

**3.2 Secondary Objective:** To evaluate the independent predictive value of other prostate cancer markers including PCA3, TMPRSS2:ERG, and 3-month finasteride PSA velocity on the performance of the PCPT risk calculator.

**Procedures:** After informed consent, eligible subjects will be randomized in a 4:1 manner (for the rationale of choosing a 4:1 randomization schema see power calculation under Specific Aim 2) to receive a bottle containing 100 tablets of finasteride 5mg or placebo and will take one tablet daily for three months. PSA will be measured monthly. Serum PSA samples will be sent to Quest Diagnostics for analysis. At baseline and at 3-months (90 days + 7 days), subjects will undergo PSA, DRE, PCA3, and TMPRSS2:ERG fusion. Post-DRE urine samples will be sent for analysis.

Specimen collection and processing for PCA3 and TMPRSS2:ERG assays: Urine samples will be collected following a routine DRE After DRE, the first 20 to 30 mL of urine are collected. Samples will be processed immediately using standardized methods of GenProbe; thereafter, they will be transferred for analysis.^[21]^ PCA3 and PSA mRNAs in post-DRE urine are quantified using transcription-mediated amplification. PCA3 copy levels are normalized to PSA mRNA; the latter functions as a prostate-specific housekeeping gene that controls for the amount of prostate cells recovered following DRE. The final output of the assay is the PCA3 Score, defined as [(PCA3 mRNA copies/mL) / (PSA mRNA copies/mL)] x 1000. The quantitative TMPRSS2:ERG assay utilizes the same specimen type, assay format and procedure as the PCA3 assay.^[22]^ The TMPRSS2:ERG mRNA isoform target corresponds to fusion between TMPRSS2 Exon1 and ERG Exon4.^[23]^ TMPRSS2:ERG mRNA copy levels are normalized to PSA mRNA to yield a TMPRSS2:ERG Score.

Specimen collection for research: Approximately two tablespoons of blood in approximately two tubes will be drawn from a vein in the participant’s arm. This blood will be used for the development of prostate cancer detection, discovery, and validation tests. One tube will be for serum and DNA and the other for plasma and dimethyl sulfoxide (DMSO) preserved white blood cells.

**Prostate Biopsy:** At 90 days +/- 14 days, subjects will undergo prostate biopsy. A prostate biopsy will be performed with the patient in the lateral decubitus position. The prostate will be imaged with transrectal ultrasound; gland is calculated for a prolate ellipse (length X width X height)(π/6). A total of 12 cores will be obtained. Two cores will be obtained from each side of the prostate at the apex, mid-gland, and base. Each core will be fixed in formalin and processed for pathologic assessment. Each of the pairs of cores will be individually read for the presence of prostate cancer (Gleason grade, cancer percent per core, perineural invasion), presence of prostatic intraepithelial neoplasia, atypia, or inflammation. After biopsy, subjects will be followed clinically using local standard of care.

A summary of the procedures is noted in the Study Calendar (Table I).

**Table I**. Study Calendar

| Study test | | Baseline | | 1 month | 2 months | | 3 months^a^ | |  |
| --- | --- | --- | --- | --- | --- | --- | --- | --- | --- |
| Consent | | X | |  |  | |  | |  |
| Serum PSA (serum)  Blood Draw (research) | | X | | X | X | | X  X | |  |
| DRE | | X | |  |  | | X | |  |
| PCA3 (urine) | | X | |  |  | | X | |  |
| ETS gene fusion (urine) | | X | |  |  | | X | |  |
| Family history assessment | | X | |  |  | |  | |  |
| Date of birth | | X | |  |  | |  | |  |
| Ethnicity/race assessment | | X | |  |  | |  | |  |
| Dispense finasteride/placebo 5mg/day | | X^c^ | |  |  | |  | |  |
| Prostate biopsy | |  | |  |  | | X^b^ | |  |
| Pill PPill Count |  | | X | | | X | | X | |
| Record Adverse Experiences | |  | | X | X | | X | |  |

^a^Final visit must be at 90 days + 7 days from study registration.

^b^Subjects must undergo prostate biopsy at 90 days + 14 days from study registration

^c^Finasteride/placebo will be dispensed within 3 business days of the Baseline Visit.

**Study Drug:** Patients will be randomized 4:1 to finasteride: placebo. Study drug will be provided by Merck & Co to the STVHCS Research Pharmacy in coded bottles specific for this study. The STVHCS Research Pharmacy will then dispense the specific container for each patient from the three sites (STVHCS, University Hospital Downtown, Robert B. Green Campus and MARC Urology Clinics). The advantage of using medication from Merck is that matching placebo will be provided.

The study drug will be delivered to the participants within 3 business days of the Baseline Visit by the research staff and/or an UTHSCSA approved delivery service.

Clinical supplies must be received by a designated person at the study site, handled and stored safely and properly, and kept in a secured location to which only the investigator and designated assistants have access. Clinical supplies are to be dispensed only in accordance with the protocol. The investigator is responsible for keeping accurate records of the clinical supplies received from Merck Sharp and Dohme Corp., the amount dispensed to and returned by the patients, and the amount remaining at the conclusion of the study.

In accordance with Good Pharmacy Practices, gloves should always be worn by study personnel if directly handling tablets or capsules that are returned (i.e., when counting returns). Women should not handle crushed or broken tablets of PROSCAR when they are or may potentially be pregnant because of the possibility of absorption of finasteride and the subsequent potential risk to a male fetus. PROSCAR tablets are coated and will prevent contact with the active ingredient during normal handling, provided that the tablets have not been broken or crushed. The investigator or designated assistant should not open individual clinical supply containers and count tablets/capsules, etc., before dispensing to the patients.

At the end of the study, all clinical supplies including partial and empty containers will be returned to Merck & Co., Inc., using the Clinical Supplies Return Form (R464). Please contact your Merck representative for review of shipment and form before shipping.

The storage conditions will be indicated on the label. The clinical supplies storage area at the site must be monitored by the pharmacy site staff for temperature consistency with the acceptable storage temperature range as specified on the label or in the product label attached to the protocol. Documentation of temperature monitoring should be maintained.

At the close of the study after un-blinding, a letter will be sent by the investigator to all subjects with their randomized drug assignment. The subjects who received placebos in the image of PROSCAR will be provided the following advice:

“You have participated in a study conducted by The University of Texas Health Science Center at San Antonio. This is to advise you that you were among those who received a look-alike tablet to resemble the drug PROSCAR (finasteride 5 mg) as much as possible. You did not receive the active drug PROSCAR (finasteride 5 mg) as manufactured by Merck & Co., Inc.”

**3.3 Procedures specific to research:**

1. Obtain signed informed consent

2. Collection of demographic information, including age, race, ethnicity, family history of prostate cancer

3. Collection of pre-biopsy urine samples to establish biomarker measurements of PCA3 and ETS gene fusion.

4. 4 to 1 Randomization to finasteride versus placebo

5. Monthly PSA lab collection

6. Pill counts

7. Collection of blood samples for prostate cancer detection, discovery,

and validation tests.

- 1. **Clinical procedures that would be done for routine care, irrespective of the research study:**

1. DRE prior to prostate biopsy
2. Prostate biopsy procedure
   1. **Subject Population**
3. **Subjects**: As this study examines prostate cancer, a disease that affects only men, only men will be enrolled. It is anticipated that the distribution of minorities in this study will reflect the minority-rich San Antonio and South Texas population. We anticipate enrolling men in the same racial/ethnic distribution as our other biomarker studies: we expect 13.5% of subjects to be African American, 37% will be Hispanic and 49.5 % Caucasian.

**Source**: Subjects will be drawn from patients seen in the Urology clinics associated with The University of Texas Health Science Center at San Antonio located at the Medical Arts Research Center (MARC), University Hospital Downtown, Robert B. Green Campus and South Texas Veterans Health Care System (STVHCS), Audie L. Murphy Division.

1. **Eligibility**

**Inclusion Criteria:**

1. Risk of prostate cancer 20-60% calculated with the on-line PCPT prostate cancer risk calculator. (www.prostate-cancer-risk-calculator.com).^[15]^ PSA value must be obtained within 3 months prior to study entry. A description of the frequency of these individuals in the population is provided in Specific Aim 2.

1. Patient has been recommended to undergo and plans to have a prostate biopsy.
2. Patient is willing to delay prostate biopsy for a 3-month finasteride treatment.
3. No allergy to finasteride or other five alpha reductase inhibitors.
4. Patient is willing to take finasteride 5 mg orally daily for 3-month treatment period.
5. Age 55 or older. (This age is selected as the PCPT risk calculator is only valid for this age range.)

**Exclusion Criteria:**

1. Risk of cancer greater than 60% or less than 20%.

1. Prior history of prostate cancer.
2. Prior treatment with finasteride or dutasteride in the past 6 months
3. Younger than age 55.
   1. **Description of Recruitment and consent Procedures**
4. **Recruitment Period:**

The study is expected to start in first half of 2010. Our recruitment experience suggests that we can reasonably accrue greater than 150 patients per year, reaching the required sample size over a 4-year study period. We anticipate enrolling 100 subjects in Year One, 150 subjects in Years Two-Three, and 100 subjects in Year Four, allowing for completion of the 3-month finasteride exposure and an analysis period. Although we perform more than 2,500 biopsies annually, eligibility criteria and patient interest will result in this accrual rate as not all patients will be willing to participate or delay the biopsy by 3 months.

1. **Identification and recruitment**

Research staff approved to work on this study will review clinic schedules of the UTHSCSA Department of Urology to identify potential subjects. Potential study candidates will be identified and the information made available to the physician who sees the patient in clinic. Physicians seeing patients in the Urology Clinics will also identify study candidates. The decision to approach identified patients about this study will be made by the physician who sees the patient. Suitable candidates will initially be approached by one of the physician investigators and invited to participate in the study. Appropriate waivers will be submitted for identification and recruitment of subjects. Participant can learn about the study through IRB approved study advertisements displayed at events, functions and clinical sites at approved study sites (UTHSCSA, MARC, UHS facilities & STVHCS), on the CTRC and Facebook websites, as well as on [http://www.clinicaltrials.gov](http://www.clinicaltrials.gov/) and [http://studies.uthscsa.edu](http://studies.uthscsa.edu/). Interested participants may contact the research staff to inquire about the research study. Eligible participants will be scheduled for a research appointment. The screening information of the potential subjects who are not enrolled into the study will be shredded and destroyed immediately.

**3. Consenting:**

The technical aspects of the study will be discussed with potential subjects by the Principal Investigator and by study physician co-investigators. The remainder of the consent process may be conducted by a research staff member who is IRB trained and designated for that role. The following consent elements will be discussed: voluntary nature of participation, purpose of the study, study procedures, risks and benefits, the collection of protected health information, confidentiality, and subject rights. The subject will be given a copy of the informed consent to read and consider and ample opportunity will be given to answer any questions. The subject may decline to participate, decide to participate, or take the consent home to think about participating. A copy of the signed consent form will be provided to each subject and a copy will be placed in the medical record.

**4.** **Location of signed consent forms and data collection**

Signed consent forms and case report forms will be stored in a locked file at the STVHCS and MARC clinic locations approved research sites. Access to these rooms is limited to designated research personnel.

**4. Human Subjects**

**4.1 Compensation and Costs**

Participants will be compensated $25.00 per visit for a total of $100.00 for participating in the study. Study drug will be provided to subjects at no cost. There will be no cost to collect or process research specific biomarkers. Patient’s health insurance company will be responsible for the costs of treatments and procedures that would be done whether or not they take part in this study, such as, digital rectal exams, and transrectal ultrasound (TRUS) with prostate biopsy.

**4.2 Risks to subject**

There is a small chance that the subject could experience bleeding or infection from the prostate biopsy. Under the proposed protocol, each study site will use their standard procedures for obtaining the prostate biopsy tissue that will consist of a minimum of 12 cores. Prophylactic antibiotics will be prescribed per site’s standard of care. There would be no psychological, legal or social risks associated with the study. Side effects with finasteride include both advantages and disadvantages. Advantages from finasteride include improved urinary stream, reduced urinary frequency and nocturia, and increased hair growth if male pattern baldness is present. Disadvantages include decreased ejaculate volume, libido, and erectile dysfunction and gynecomastia. These adverse experiences generally did not lead to discontinuation of the study drug. Finally, there is potential risk that a delay in diagnosis of prostate cancer by as much as 3 months could affect the outcome of treatment. Evidence would suggest that this risk is low, especially due to the risk range selected for study and the fact that most prostate cancers develop over a period of years to decades.

[Information from the U.S. Food and Drug Administration (FDA) Drug Safety Communication on June 9, 2011 resulted in the following text added to the informed consent form] - Finasteride was studied in a clinical trial of almost 19,000 men, sponsored by the National Cancer Institute. The study was designed to determine if the drug could prevent the development of prostate cancer. The final results of the study showed that finasteride, taken over 7 years, reduced a man’s risk of prostate cancer by about 25%. It also found an increase in detection of a less-common, more aggressive type of prostate cancer. While it is possible that this medication may cause this more aggressive tumor, it was also found that in men who were taking finasteride, detection tests (PSA and prostate rectal examination) and prostate biopsy more easily detected the aggressive tumors which were more commonly missed in men who were not taking the medication. It is this improved detection of prostate cancer that it is hoped will be seen in this 3-month period.

**4.3 Special precautions: Procedures for** **Minimizing Risks, Treating Adverse Effects to Ensure Subject Safety**

Risks of phlebotomy: risks will be minimized by using universal precautions and standard clinical procedures for phlebotomy. Blood draws will be performed by appropriately trained personnel who pay careful attention to technique and abide by established standards (use of sterile needles, blood and body fluid precautions); ensure that tubes are within their expiration date; and study protocol procedures are followed.

Prostate Tissue Collection: Risks are minimized by using visits in conjunction with Standard of Care. Biopsy procedures will be completed by study physician who is credentialed to perform this procedure. Appropriate protocol technique will be utilized with care taken to minimize risks. Privacy will be offered as per standard care. Subject data will be examined and subjects queried for adverse events. The data will be periodically reviewed by the Principal Investigator to ensure quality control and safety.

**4.4 Alternative Treatments**

Patients who are eligible for the proposed study will be receiving the current standard of care: prostate biopsy. In general, prostate biopsy would be offered at a next available time or depending on the patient’s schedule. On average, the time between initial consultation for biopsy and the biopsy itself may range from 4-6 weeks or longer. As such, the delay for prostate biopsy by study participation would be in the range of 6-8 weeks.

**4.5 Confidentiality**

Protected Health Information may be shared with the researchers at The University of Texas Health Science Center at San Antonio (UTHSCSA). The Institutional Review Board (IRB) and Compliance Office of the University of Texas Health Science Center at San Antonio, representatives of the University Hospital System, Research Office,and South Texas Veterans Health Care System (STVHCS) Hospital, Audie L. Murphy Division, Research & Development and Compliance Offices. In addition, representatives of other groups that have the responsibility of monitoring and overseeing research studies, and the Food and Drug Administration (FDA). Maintaining confidentiality of patient-specific information will be top priority throughout all phases of the study. Patient data (PHI) will be compiled in a database and de-identified upon completion of analysis. Cases in the database will be identified by initials, patient identification number, the year of patient’s birth, and the date of biopsy. The database will not include information which can be identifiable with the link (or key). All electronic data will be stored in a password protected database in accordance with UTHSCSA computer-security policies. The identification number is actually a research record number that cannot be linked to the subject except by a key. This key will be maintained by the research staff in the enrollment log stored on a password protected UT Server. Informatics Data Exchange and Acquisition System (IDEAS) designed and maintained by the Department of Epidemiology and Biostatistics (DEB) will be used to manage data and monitor data entry. Identification of any specimens will be by patient study number assigned at time of trial entry. Parts of collected PHI may be photocopied and sent to a central location or may be transmitted electronically, such as by e-mail or fax.

**5. Safety Monitoring**

**5.1** **Adverse Events**

An adverse event (AE) is the appearance of (or worsening of any pre-existing) undesirable sign(s), symptom(s), or medical condition(s) occurring after a participant signs the informed consent even if the event is not considered to be related to the study. An AE can therefore be any unfavorable and unintended sign (including abnormal laboratory finding), symptom, or disease temporally associated with any study-related procedure or the use of study medication, whether or not considered related to the study-related procedure or the use of study medication.. This definition includes inter-current illnesses or injuries, and exacerbation of pre-existing conditions. Indicators of disease progression will not be reported as AEs. Any abnormal clinical test results (PSA, BMP and urinalysis) or other safety assessments (radiological scans or vital signs) that are associated with the underlying disease, unless judged by the investigator to be more severe than expected for the subject’s condition, are not to be reported as AE’s.

Adverse events that are directly related to research specific procedures (phlebotomy and prostate biopsy) will be graded for severity according to the NCI Common Terminology Criteria for Adverse Events (CTCAE), version 3.0 (available online at <http://ctep.cancer.gov/reporting/ctc.html>). If CTCAE grading does not exist for an AE, the grading descriptor of mild, moderate, severe, life-threatening, and death, or grades 1-5, will be used.

**5.2 Serious Adverse Events**

A serious adverse event (SAE) is any untoward medical occurrence that:

1. Is fatal or life-threatening

2. Requires hospitalization or prolongation of existing hospitalization

3. Results in disability/incapacity

4. Is medically significant in that it may jeopardize the patient and may require medical or surgical intervention to prevent one of the outcomes listed above.

**5.3 Collecting** **and Recording Adverse Events**

Any AE or SAE occurring during the study must be documented in the subject’s medical record in accordance with the investigator’s normal clinical practice and on the AE or SAE case report form (CRF) for the study. An adverse event that occurs after study consent is signed but before research-related procedures will be recorded as medical history.

**5.4 Assessment of Causality**

The investigator is obligated to assess the relationship between any study-related procedure or study medication and the occurrence of each AE/SAE. The investigator will use clinical judgment to determine the relationship. Alternative causes, such as natural history of the underlying diseases, concomitant therapy, other risk factors, and the temporal relationship of the event to any study-related procedure or study medication will be considered and investigated.

Even in situations when an SAE has occurred and the investigator has incomplete information to include in the initial SAE report, the investigator will make an assessment of causality for every event prior to reporting it. The investigator may change his opinion of causality in light of follow-up information and amend the SAE case report form and report accordingly.

Adverse events will be reported annually to the IRB. SAEs meeting the IRB definition of Unanticipated Problems Involving Risk to Subjects or Others (UPIRSO) will be reported to the IRB within 7 days and within 48 hours if life-threatening or fatal. As Merck & Co will be providing finasteride and placebo, SAEs will also be reported to Merck within the same period. In addition, copies of all serious adverse experiences, which are possibly, probably, or definitely related to use of a Merck Product should be sent to Merck (Attn: Worldwide Product Safety; FAX 215 993-1220) within two working days. Additionally, any report of pregnancy occurring in association with use of a Merck Product should be sent to Merck (Attn: Worldwide Product Safety; FAX 215 993-1220).

**6. Data Analysis**

**6.1 Statistical Considerations for the Study**

Statistical endpoints of the study and definitions of terms are first provided for reference for the statistical plan for Specific Aims 2-5. Lists of analysis plans and power calculations follow for the required sample size of 500 (144 expected cancer cases in finasteride arm; 216 expected controls in finasteride arm; 90 subjects in placebo arm; 10% inflation for drop-out).

**Primary endpoint**:

- Operating characteristics (AUC, sensitivity and specificity) of 3-month finasteride PSA velocity for biopsy outcome.

**Secondary endpoints**:

- 3-month finasteride PSA velocity in cases and controls.
- Operating characteristics of initial and 3-month finasteride PSA, DRE, PCA3, and TMPRSS2:ERG (TMPRSS2:ERG) for biopsy outcome.

Definitions:

- Intermediate risk: risk of prostate cancer between 20% and 60% using PCPT Risk Calculator.
- 3-month finasteride PSA velocity: monthly change in log PSA while on finasteride. Clinical PSA baseline value (obtained within 3 months period prior to enrollment) and Research PSA Baseline (first) value is immediately prior to finasteride.
- Baseline (initial) PSA (DRE, PCA3, and TMPRSS2:ERG): PSA (DRE, PCA3, and TMPRSS2:ERG) value immediately prior to randomization.
- 3-month finasteride PSA (DRE, PCA3, and TMPRSS2:ERG): PSA (DRE, PCA3, and TMPRSS2:ERG) value at 3 months after 3-month of finasteride.
- 3-month placebo PSA (DRE, PCA3, and TMPRSS2:ERG): PSA (DRE, PCA3, and TMPRSS2:ERG) value at 3 months after 3-month of placebo.
- Sensitivity: The proportion of cancer cases with marker value exceeding a cutoff (specific to the choice of cutoff selected).
- Specificity: The proportion of control cases with marker value at or below a cutoff (specific to the choice of cutoff selected). Equals 100%-False Positive Rate.
- ROC curve: A graph of 1-Specificity (i.e. False Positive Rate) on the x-axis by Sensitivity on the y-axis. The closer the curve comes to the 45-degree diagonal of the ROC space, the less accurate the test.
- AUC curve: Area underneath the ROC curve ranging from 0 to 1, higher AUC indicates a better marker. Because a completely random guessing produces the diagonal line between (0, 0) and (1, 1), which has an AUC of 0.5, no realistic classifier should have an AUC less than 0.5.

*Possible Pitfalls and Alternative Strategies*:

As noted below in our statistical section, the statistical power of this study will rely on having a minimum of 144 events (prostate cancer cases) in finasteride arm to evaluate the 3-month finasteride ‘challenge’ test as an adjunct to prostate cancer detection. We expect a positive biopsy rate of 40% in men with intermediate risk of prostate cancer but will increase accrual as needed to reach this minimum number of events. We anticipate that some men may ‘drop out’ of the study’s participation due to perceived problems with the medication (e.g., perceived sexual dysfunction^[56]^) or if they simply change their mind related to the prostate biopsy. We plan on regular follow-up with all participants to keep them abreast of the study’s progress as well as new developments in prostate cancer detection. We have found that these regular follow-up telephone calls help maintaining subject interest in the trial. We have made conservative expectations on subject recruitment from our large Urology practice for accrual and do not anticipate problems reaching this number of events within the time of the study.

**6.2. Specific Aim 2**: To evaluate serum PSA velocity after a 3-month finasteride 'challenge' as a biomarker for prostate cancer on biopsy.

*Rationale: This aim is first needed to confirm that under finasteride, PSA indeed dropped more for non-cases than for subsequent cancer* cases (PSA velocity differed between cases and non-cases) *and that on its own has good (comparable to PSA alone) operating characteristics (sensitivity, specificity, and AUC).*

**6.2.1. Power estimates for Specific Aim 2**

Sample size is selected to ensure sufficient accuracy for characterizing the operating characteristics (sensitivity and specificity) of 3-month finasteride PSA velocity and to have adequate power to detect the difference in PSA decline between cases and controls (non-cases). We estimate a positive biopsy rate of 40% in study subjects. Thus, the ratio of cancer cases to non-cases (controls) is 0.67(=0.4/0.6). With finasteride, a decline of 34% was seen in controls by 3 months^[6]^; a decline of 10-15% in cancer cases is assumed, corresponding to an additive change on a logarithmic PSA scale (in order to assume normality) of -log(1-0.34)=0.416 for controls, and -log(1-0.10)=0.105 to –log(1-0.15)=0.163 for cases. An estimate of the standard deviation of log PSA from the 3379 SABOR cohort is .89. Assuming a correlation of .5 between two PSA measures from the same patient (before and after finasteride) implies the same standard deviation for the difference or change in log PSA over 3-months. With these assumptions, a sample of 360 (144 cancer cases and 216 controls) in finasteride arm achieves a 75% to 90% power to test the null hypothesis that there is no difference between cases and controls in 3-month finasteride PSA change with a significance level (alpha) of 0.05 using a two-sided two-sample t-test (Table IX).

Table IX: Power for 216 Controls (no PCa) and 144 Cases (PCa) in Finasteride Arm

| PSA decline in | PSA decline in cancer cases | | | | |
| --- | --- | --- | --- | --- | --- |
| Controls | 8 % | 10% | 12% | 14% | 16% |
| 30 % | **.814** | **.747** | .665 | .574 | .479 |
| 32 % | **.884** | **.833** | **.766** | .687 | .598 |
| 34 % | **.934** | **.900** | **.851** | **.788** | .713 |
| 36 % | **.966** | **.944** | **.912** | **.867** | **.809** |
| 38 % | **.984** | **.973** | **.954** | **.926** | **.886** |
| 40 % | **.994** | **.988** | **.979** | **.963** | **.939** |

To minimize bias and eliminate the effects of unknown confounding variables on the effect of 3-month finasteride treatment in PSA change, a blinded placebo arm is included. The Medical Therapy of Prostatic Symptoms Research Group study^[16]^, found that, among 670 men in placebo group, at year 1, the serum PSA level remained the same on average (the median change from baseline to year 1 was equal to 0). We thus anticipate that PSA will be unchanged in placebo arm for a 3-month period (i.e., average change is 0 in logPSA). With finasteride, weanticipate that the average decline in PSA is between 24.4% (=34%X0.6+10%X0.4) and 26.4% (=34%X0.6+15%X0.4), corresponding to an additive change of 0.28 (=-log[1-0.244]) to 0.31 (=1-log[1-0.264]) on a logPSA scale. We thus assume the average change is 0.3 in logPSA for finasteride arm. A sample of 450 (360 in finasteride arm and 90 in placebo arm) achieves an 81% power to detect a 0.3 difference in average logPSA change between finasteride and placebo with a type I error of 0.05 and an estimated standard deviation of 0.89 using a two-sided two-sample t-test. A 4:1 ratio of randomization scheme is a trade-off between a feasible sample size and adequate statistical power to detect a 0.3 difference in logPSA between finasteride and placebo (Table X).

**Table X:** Sample Size and Corresponding Power

| Sample size (finasteride) | Sample size (placebo) | Allocation ratio | Power |
| --- | --- | --- | --- |
| 360 | 36 | 0.1 (10:1) | 0.48 |
| 360 | 54 | 0.15 (6.67:1) | 0.63 |
| 360 | 72 | 0.2 (5:1) | 0.74 |
| **360** | **90** | **0.25 (4:1)** | **0.81** |
| 360 | 108 | 0.3 (3.33:1) | 0.86 |
| 360 | 126 | 0.35 (2.86:1) | 0.90 |

Our clinical experience suggests a 10% dropout for this three-month trial. The sample size is inflated to 500(=400/0.9). In SABOR, 1033 out of 3379 (30.6%) participants have an intermediate risk of prostate cancer.^[44]^ Therefore, in order to achieve a sample of 400 eligible intermediate risk participants, we will need to screen 1308(=400/30.6%) men. We will accrue not only from SABOR subjects but from general Urology patients in whom over 1500 biopsies are performed annually.

Our sample size calculation is based on the expected sensitivity (or specificity) and the desired precision of the confidence interval (CI) for the sensitivity (or specificity). Our primary interest is whether a 3-month finasteride PSA velocity has a low false positive rate (high specificity) to reduce unnecessary biopsy for a fixed sensitivity. It is thus appropriate to estimate sensitivities less precisely than specificities. Our analysis^[12]^ (1225 cancer cases vs. 4362 controls) found sensitivities ranging from 20 to 40% for specificities ranging from 80 to 95%. With 144 cancer cases, using the binomial formula, the standard error for sensitivities can be controlled within 4.1%. Similarly, with 216 controls, the standard error for specificities can be controlled within 2.7%.

**6.2.2. Analysis for Specific Aim 2**

Three-month finasteride/placebo PSA velocity will be determined for each participant by linear regression of the logarithmically transformed PSA using all four PSA values at baseline, 1, 2, and 3 months. The slope of the fitted regression line is the empirical estimate of 3-month finasteride/placebo PSA velocity for each individual. Differences between cases and controls will be accessed via a two-sample t-test on these slopes at the alpha=.05 level of statistical significance for finasteride and placebo arms separately. Sensitivities and specificities for 3-month finasteride/placebo PSA velocity will be calculated according to a range of cutoffs of velocity. For this application, lower values of velocity, generally negative in sign, (more steep declines) correspond to controls and higher values, generally positive in sign, (less declines) correspond to cases. The area underneath the receiver operating characteristic curve and corresponding 95%CI will be calculated. A test of null hypothesis of no predictive information (AUC=0.5) will be performed using Wilcoxon rank test and U statistics at the alpha=.05 level of statistical significance.^[44]^

Table III showed that PSA has better performance characteristics for the detection of high-grade disease than for overall risk of prostate cancer. Therefore, in our study, we plan to explore the operating characteristics of 3-month finasteride PSA velocity specifically for higher-grade (Gleason ≥ 7) cancers. The same test statistics (AUC, sensitivity and specificity) will be calculated as described before.

*Potential pitfalls and alternative strategies for Specific Aim 2*

The distribution of 3-month finasteride PSA velocity in cases and controls may not appear to be normally distribution by inspection of histograms, violating the Normal assumption required for the two-sample t-test. In this case the less powerful but robust nonparametric Mann-Whitney test will be used to compare the distributions. The definition of 3-month finasteride PSA on the log scale and corresponding cutoff are cumbersome to interpret because the log transformation is a nonlinear transformation. Therefore simpler more interpretable definitions of velocity will be evaluated and compared. One definition we have recently suggested is the percent change in PSA from baseline. This definition is less precise as it uses the first and last PSA measure as opposed to all PSA measures at each month as used by our proposed definition. Missing PSA observations will be a potential concern although we expect a low incidence. For individuals with only the first and last PSA available, 3-month finasteride PSA velocity can be alternatively defined as the difference in logPSA values divided by the difference in time points. The 3-month PSA endpoint will not be available from individuals who dropped out resulting in inability to make comparisons to the primary endpoint of PSA velocity and a reduction of sample size. In anticipation of incompleters, our targeted sample size of 360 subjects is inflated by 10%, for a total of 400 enrollees.

**6.3.** **Specific Aim 3**: To characterize the operating characteristics of serum PSA and DRE before and after 3 months of finasteride and compare to the 3-month finasteride ‘challenge’ as markers for disease.

*Rationale: We have advocated that newly proposed biomarkers should not be examined in isolation but rather calibrated against standard approaches, especially when the standard is cheaper and/or easier to measure. A single PSA is easier to use than computation of PSA velocity. Because of the cohort-dependent effect on PSA operating characteristic studies, we further emphasize that the comparison be made on the same cohort.*

**6.3.1. Power for Specific Aim 3**

The operating characteristics of 3-month finasteride PSA velocity will be compared to four additional markers: initial (before finasteride) PSA, 3-month finasteride PSA (current PSA value after 3 months on finasteride), initial DRE, and 3-month finasteride DRE (DRE result after 3 months on finasteride) on the same series of patients. The same power calculation as for Specific Aim 2 applies: sensitivities and specificities of these markers in the ranges 20 to 40% and 80 to 95%, respectively, can also be estimated with the same degree of accuracy (standard error within 4.1% for sensitivity; 2.7% for specificity) from 144 cases and 216 controls.

Based on the unbalanced sample size, tests of the null hypotheses that sensitivities and specificities of 3-month finasteride PSA velocity equals that of these alternative markers will obtain sufficient (80%) power to detect small elevations in specificities but relatively large elevations in sensitivities. This disadvantage is alleviated because we are primarily interested in the effect of 3-month finasteride PSA on increasing specificity. Specifically, with 144 cancer cases, the study will have 80 percent power to detect an increase of 13.5, 15.8 and 17.8 percentage points in sensitivity of 3-month finasteride PSA velocity relative to the sensitivities of 20%, 30% and 40% of the alternative markers, respectively. Conversely, with 216 controls, the study will obtain 80% power to detect increases of 8.6, 7.4, 6.1 and 4.3 percentage points in specificity of 3-month finasteride PSA velocity relative to the specificities of the other markers when the specificities equal 80%, 85%, 90% and 95%, respectively. The power calculations are based on the two-sided McNemar’s test with significance level of 0.05 using the midpoint method proposed by Lachenbruch(1992).^[66]^

**6.3.2. Analysis for Specific Aim 3**

Pre-finasteride PSA and DRE and 3-month finasteride PSA and DRE will be considered as four additional markers to 3-month finasteride PSA velocity measured on the same subject. Sensitivities, specificities and AUCs (excluding the dichotomous test DRE for which the AUC cannot be computed) of these markers will be determined and compared against each other and 3-month finasteride PSA velocity. Tests of differences in the AUC between all pairs of markers (excluding DRE) will be performed using the U-statistic approach of DeLong et al., which controls for the dependencies among individuals.^[67]^ Marker sensitivities and specificities will be compared using McNemar’s test. Comparisons will be made in 2 fashions. First, the marker sensitivities will be compared at matched-specificities, e.g., cut-offs for each marker will be found which obtain 80% specificity and then sensitivities at these cutoffs will be compared. Second, and of more interest in the application, marker specificities will be compared at matched-sensitivities. We hypothesize that 3-month finasteride PSA velocity will have increased specificity (smaller false positive rate) for a given sensitivity than the other markers.

*Potential pitfalls and alternative strategies for Specific Aim 3*

The 3-month PSA and DRE endpoint will not be available from individuals who dropped out resulting in inability to make comparisons to the primary endpoint of PSA velocity and a reduction of sample size. In anticipation of incompleters, our targeted sample size of 360 subjects is inflated by 10%, for a total of 400 enrollees.

**6.4. Specific Aim 4**: To assess the independent diagnostic value of 3-month finasteride serum PSA velocity to the Prostate Cancer Prevention Trial (PCPT) prostate cancer risk calculator.

*Rationale: Extending the rationale of Specific Aim 2, we have observed that new biomarkers may not add independent prognostic value to established risk factors including family history of disease or age, in addition to standard biomarkers. Hence the ultimate goal of this study is to establish the usefulness of 3-month finasteride PSA as an independent marker to all established biomarkers and risk factors for prostate cancer.*

**6.4.1. Analysis for Specific Aim 4**

Multivariable logistic regression will be used to ascertain whether 3-month finasteride PSA velocity adds independent diagnostic information for predicting cancer outcome on biopsy relative to the PCPT risk factors: PSA, DRE, family history and prior biopsy on this N=360 cohort. 3-month finasteride PSA velocity will be declared to add independent diagnostic information to the risk factors if it retains statistical significance at the 0.05 level when included in the multivariable logistic regression along with all other risk factors. This particular analysis may have limited power since the cohort is relatively small and several risk factors will be estimated.

An analysis with higher power is obtained by our recent methodology for incorporating a new marker from an external study into the PCPT risk calculator through computation of the likelihood ratio.^[24]^ The likelihood ratio of 3-month finasteride PSA velocity will be defined as the ratio of probabilities of observing a specific 3-month finasteride PSA velocity value in cases relative to controls stratified by the PCPT risk factors. The probabilities will be modeled separately for cases and controls by linear regression with 3-month finasteride PSA velocity as outcome and covariates the PCPT risk factors: race, age, log PSA (before finasteride), DRE (before finasteride), family and prior biopsy history. By Bayes formula, an updated posterior odds of prostate cancer given 3-month finasteride PSA velocity and the PCPT risk factors is equal to the product of the likelihood ratio by the prior odds defined by the PCPT risk calculator. The posterior odds can be transformed into the posterior risk by posterior risk = posterior odds/[1+posterior odds]. ^(1)^

Graphs of the posterior risk against the prior risk will be examined to assess the potential impact of 3-month finasteride PSA velocity as an additional risk factor to the PCPT risk factors.

*Potential pitfalls and alternative strategies for Specific Aim 4*

The standard deviations in the likelihood ratios may be high resulting in updated PCPT risks with large confidence bands (high uncertainty). These confidence bands will be displayed and reported. In this case, the need for further validation studies of 3-month finasteride PSA as a biomarker will be needed.

**6.5. Specific Aim 5:** To assess the independent predictive value of additional prostate cancer biomarkers including PCA3 and a ETS gene fusion (TMPRSS2:ERG) on the performance of the PSA-based markers and, in combination or in place of the results of the finasteride ‘challenge’ test.

*Rationale: Among a host of new biomarkers for prostate cancer, these three markers are among the most promising. This Aim will determine if they add to the performance of baseline risk assessment (using PSA and baseline demographics alone – the PCPT risk calculator), if they are superior to the finasteride ‘challenge’ results, and whether they further improve on the results of the finasteride ‘challenge’.*

**6.5.1. Study design**: At baseline, all men will undergo a DRE in which a minimum of 3 strokes are made on each lobe of the prostate after which a urine sample is obtained. After samples are obtained, one will be processed for PCA3 and the other for TMPRSS2:ERG. All samples will be de-identified at UTHSCSA prior to shipment.

**6.5.2. Sample Collection.**  Urine samples will be shipped to GenProbe for PCA3 and TMPRSS2:ERG analysis in a blinded fashion.

Specimen collection and processing for PCA3 and TMPRSS2:ERG assays: Urine samples will be collected following a DRE by applying firm pressure with several strokes to the prostate. After DRE, the first 20 to 30 mL of urine will be collected. Samples will be processed immediately using standardized methods of GenProbe; thereafter, they will be transferred to GenProbe for analysis.^[23]^ PCA3 and PSA mRNAs in post-DRE urine are quantified using transcription-mediated amplification. PCA3 copy levels are normalized to PSA mRNA; the latter functions as a prostate-specific housekeeping gene that controls for the amount of prostate cells recovered following DRE. The final output of the assay is the PCA3 Score, defined as [(PCA3 mRNA copies/mL) / (PSA mRNA copies/mL)] x 1000. The quantitative TMPRSS2:ERG assay utilizes the same specimen type, assay format and procedure as the PCA3 assay.^[37]^ The TMPRSS2:ERG mRNA isoform target corresponds to fusion between TMPRSS2 Exon1 and ERG Exon4.^[26]^ TMPRSS2:ERG mRNA copy levels are normalized to PSA mRNA to yield a TMPRSS2:ERG Score.

Results of all analyses will be transmitted to Dr. Liang at UTHSCSA for analysis.

**6.5.3. Key objectives**

To achieve Specific Aim 5, the following key objectives are identified.

- Objective 1: To assess whether any of the biomarkers (PCA3 and TMPRSS2:ERG) are superior to the PCPT risk calculator (RC) for estimating the risk of prostate cancer.
- Objective 2: To assess whether any of the biomarkers (PCA3 and TMPRSS2:ERG) are superior to the 3-month finasteride PSA velocity for estimating the risk of prostate cancer.
- Objective 3 : To assess whether any of the biomarkers (PCA3 and TMPRSS2:ERG) are superior to the posterior risk calculated in Specific Aim 4 Equation (1) (i.e., a combination of PCPT RC and 3-month finasteride PSA velocity).
- Objective 4: To evaluate the independent predictive value of additional prostate cancer biomarkers including PCA3 and TMPRSS2:ERG and 3-month finasteride PSA velocity on the performance of the PCPT risk calculator.

**6.5.4. Analysis for key objectives**

- Objectives 1 to 3

Tests of differences in the AUC between all pairs of markers (Objective 1: PCPT RC vs. PCA3 and PCPT RC vs. TMPRSS2:ERG; Objective 2: PSA velocity vs. PCA3, PSA velocity vs. TMPRSS2:ERG; Objective 3: posterior risk vs. PCA3, posterior risk vs. TMPRSS2:ERG) will be performed using the U-statistic approach of DeLong et al.^[67]^ Marker sensitivities will be compared at matched-specificities; marker specificities will be compared at matched-sensitivities. The same power calculation as for Specific Aim 3 applies.

- Objective 4

Multivariable logistic regression will be used to ascertain whether 3-month finasteride PSA velocity, PCA3 and TMPRSS2:ERG add independent diagnostic information for predicting cancer outcome on biopsy relative to the PCPT risk factors in this cohort. A new biomarker will be declared to add independent diagnostic information if it retains statistical significance at the .05 level when included in the multivariable logistic regression along with all other risk factors. This analysis may have limited power since the cohort is relatively small and several risk factors will be estimated.

In addition, the approach of Ankerst et al. (2008)^[24]^ will be used to combine the predictive information of the new biomarkers. In Specific Aim 4, we plan to upgrade the PCPT risk calculator by including the 3-month finasteride PSA velocity. In Specific Aim 5, four types of updates of the PCPT risk calculator will be examined: 1) adding a single marker (PCA3, TMPRSS2:ERG, , or PSA velocity); 2) adding two markers at a time (6 possibilities); 3) adding three markers at a time (4 possibilities); and 4) adding all four biomarkers. In each case, likelihood ratios for the biomarkers will be modeled using normal distribution (for Type 1 update) or multi-normal distribution (for Type 2-4 updates) for inclusion of new biomarkers with means conditional on PCPT risk factors. The likelihood ratios will be multiplied by the prior odds of prostate cancer of the PCPT risk calculator to form the posterior risks of prostate cancer. Risk curves and surfaces will be examined to assess the potential impact of new biomarkers as additional risk factors to the PCPT risk factors for Type 1 update and Type 2-4 updates, respectively.

*Potential pitfalls and alternative strategies for Specific Aim 5*

As it is the primary focus, this study is mainly designed to have adequate power for Specific Aim 2 and 3, and the first three objectives of Specific Aim 5. The standard deviations in the likelihood ratios may be high resulting in updated PCPT risks with large confidence bands (high uncertainty). In this case, the observed data will assist us in planning a larger study in the future to further evaluate the independent predictive value of additional prostate cancer biomarkers and to improve the confidence interval estimations of the posterior risks of prostate cancer. A major advantage of this study design is that urine and serum samples will be reserved in the UTHSCSA biorepository. As biomarkers are discovered, it will be possible to retrospectively query study samples to address the same questions as are prospectively planned in this study for these new markers.

**7. Literature Cited.**

1. Kramer BS, Hagerty KL, Justman S, Somerfield MR, Albertsen PC, Blot WJ, Carter HB, Costantino JP, Epstein JI, Godley PA, Harris RP, Wilt TJ, Wittes J, Zon R, Schellhammer P. Use of 5 alpha reductase inhibitors for prostate cancer chemoprevention: American Society of Clinical Oncology/American Urological Association 2008 Clinical Practice Guideline. J Clin Onc, In press.
2. Epstein JI. What’s new in prostate cancer disease assessment in 2006? Curr Opin Urol 2006;16:146-51.
3. Jemal A, Siegel R, Ward El, Murray T, Xu J, Thun MJ. Cancer Statistics 2007. CA Cancer J Clin 2007;57:43-66.
4. Thompson IM, Ernst JJ, Gangai MP, Spence CR. Adenocarcinoma of the prostate: results of routine urological screening. J Urology 1984;132:690-2.
5. Weir HK, Thun MJ, Hankey BF, Ries LA, Howe HL, Wingo PA, Jemal A, Ward E, Anderson RN, Edwards BK. Annual report to the nation on the status of cancer, 1975-2000, featuring the uses of surveillance data for cancer prevention and control. J Natl Cancer Inst 2003;95:1276-99.
6. Sirovich BE, Schwartz LM, Woloshin S. Screening men for prostate cancer colorectal cancer in the United States: does practice reflect the evidence? JAMA 2003;289:1414-20.
7. Hodge KK, McNeal JE, Stamey TA. Ultrasound guided transrectal core biopsies of the palpably abnormal prostate. J Urology 1989;142:66-70.
8. [Roehl KA, Antenor JA, Catalona WJ.](http://www.ncbi.nlm.nih.gov/sites/entrez?Db=pubmed&Cmd=ShowDetailView&TermToSearch=12187191&ordinalpos=15&itool=EntrezSystem2.PEntrez.Pubmed.Pubmed_ResultsPanel.Pubmed_RVDocSum) Robustness of free prostate specific antigen measurements to reduce unnecessary biopsies in the 2.6 to 4.0 ng./ml. range. J Urol. 2002;168:922-5.
9. [Thompson IM, Goodman PJ, Tangen CM, Lucia MS, Miller GJ, Ford LG, Lieber MM, Cespedes RD, Atkins JN, Lippman SM, Carlin SM, Ryan A, Szczepanek CM, Crowley JJ, Coltman CA Jr](http://www.ncbi.nlm.nih.gov/pubmed/12824459?ordinalpos=28&itool=EntrezSystem2.PEntrez.Pubmed.Pubmed_ResultsPanel.Pubmed_RVDocSum). The influence of finasteride on the development of prostate cancer. N Engl J Med. 2003;349:215-24.
10. [Thompson IM, Pauler DK, Goodman PJ, Tangen CM, Lucia MS, Parnes HL, Minasian LM, Ford LG, Lippman SM, Crawford ED, Crowley JJ, Coltman CA Jr](http://www.ncbi.nlm.nih.gov/sites/entrez?Db=pubmed&Cmd=ShowDetailView&TermToSearch=15163773&ordinalpos=1&itool=EntrezSystem2.PEntrez.Pubmed.Pubmed_ResultsPanel.Pubmed_RVDocSum). Prevalence of prostate cancer among men with a prostate-specific antigen level < or =4.0 ng per milliliter. N Engl J Med. 2004;350:2239-46.
11. [Thompson IM, Ankerst DP, Chi C, Lucia MS, Goodman PJ, Crowley JJ, Parnes HL, Coltman CA Jr.](http://www.ncbi.nlm.nih.gov/sites/entrez?Db=pubmed&Cmd=ShowDetailView&TermToSearch=15998892&ordinalpos=3&itool=EntrezSystem2.PEntrez.Pubmed.Pubmed_ResultsPanel.Pubmed_RVDocSum) Operating characteristics of prostate-specific antigen in men with an initial PSA level of 3.0 ng/ml or lower. JAMA. 2005;294:66-70.
12. Thompson IM, Ankerst DP, Chi C, Goodman PJ, Tangen CM, Lucia MS, Feng Z, Parnes HL, Coltman CA. Assessing prostate cancer risk: results from the Prostate Cancer Prevention Trial. J National Cancer Institute 2006;98:529-34.
13. [Leman ES, Cannon GW, Trock BJ, Sokoll LJ, Chan DW, Mangold L, Partin AW, Getzenberg RH](http://www.ncbi.nlm.nih.gov/sites/entrez?Db=pubmed&Cmd=ShowDetailView&TermToSearch=17445657&ordinalpos=2&itool=EntrezSystem2.PEntrez.Pubmed.Pubmed_ResultsPanel.Pubmed_RVDocSum). EPCA-2: a highly specific serum marker for prostate cancer. Urology. 2007;69:714-20.
14. [Meiers I, Shanks JH, Bostwick DG.](http://www.ncbi.nlm.nih.gov/sites/entrez?Db=pubmed&Cmd=ShowDetailView&TermToSearch=17558856&ordinalpos=2&itool=EntrezSystem2.PEntrez.Pubmed.Pubmed_ResultsPanel.Pubmed_RVDocSum) Glutathione S-transferase pi (GSTP1) hypermethylation in prostate cancer: review 2007. Pathology. 2007;39:299-304.
15. [Marks LS, Fradet Y, Deras IL, Blase A, Mathis J, Aubin SM, Cancio AT, Desaulniers M, Ellis WJ, Rittenhouse H, Groskopf J.](http://www.ncbi.nlm.nih.gov/sites/entrez?Db=pubmed&Cmd=ShowDetailView&TermToSearch=17382159&ordinalpos=3&itool=EntrezSystem2.PEntrez.Pubmed.Pubmed_ResultsPanel.Pubmed_RVDocSum) PCA3 molecular urine assay for prostate cancer in men undergoing repeat biopsy. Urology. 2007;69:532-5.
16. Guess HA, Heyse JF, Gormley GJ. The effect of finasteride on prostate-specific antigen in men with benign prostatic hyperplasia. Prostate 1993;22:31-7.
17. Bussemakers MJG, van Bokhoven A, Debruyne FMJ, Isaacs WB. DD3: A new prostate-specific marker, strongly overexpressed in prostatic tumors [Abstract #83]. Journal of Urology 1997;157: 21.
18. Bussemakers MJG, van Bokhoven A, Verhaegh GW, Smit FP, Karthaus HFM, Schalken JA, Debruyne FMJ, Ru N, Isaacs WB. DD3: A new prostate-specific gene, highly overexpressed in prostate cancer. Cancer Research 1999; 59: 5975-9.
19. Hessels D, Gunnewiek JMTK, van Ooort I, Karthaus HFM, Van Leenders GJL, Van BaLKEN b, Kiemeney LA, Wities JA, Schalken JA. DD3^PCA3^-based molecular urine analysis for the diagnosis of prostate cancer. European Urology 2003; 44: 8-16.
20. Landers KA, Burger MJ, Tebay MA, Purdie DM, Scells B, Samaratunga H, Lavin MF, Gardiner RA. Use of biomarkers for a molecular diagnosis of prostate cancer. International Journal of Cancer 2005; 114: 950-6.
21. Verhaegh GW, van Bokhoven A, Smit FP,Schalken JA, Bussemakers MJG. Isolation and characterization of the promoter of the human prostate cancer-specific DD3 gene. Journal of Biochemistry 2000; 275: 37496-503.
22. de Kok JB, Verhaegh GW, Roelofs RW,Hessels D, Kiemeney LA, Aalders TW, Swinkels DW, Schalken JA. DD3^PCA3^, a very sensitive and specific marker to detect prostate tumors. Cancer Research 2002; 62: 2695-8.
23. Groskopf J, Aubin SMJ, Deras IL,Blase A, Bodrug S, Clark C, Brentano S, Mathis J, Pham J, Meyer T, Cass M, Hodge P, Macairan ML, Marks LS, Rittenhouse H. APTIMA PCA3 molecular urine test: Development of a method to aid in the diagnosis of prostate cancer. Clinical Chemistry 52: 1089-95, 2006.
24. Ankerst DP, Groskopf J, Day JR, Blasé A, Rittenhouse H, Pollock BH, Tangen CM, Parekh D, Leach R, Thompson IM. Predicting prostate cancer risk through incorporation of a molecular biomarker PCA3. Journal of Urology 2008; 180: 1303-8. (PMID: 18707724)
25. Nakanishi H, Groskopf J, Fritsche HA, Bhadkamkar V, Blase A, Kumar SV, Davis JW, Troncoso P, Rittenhouse H, Babaian RJ. PCA3 molecular urine assay correlates with prostate cancer tumor volume: implication in selecting candidates for active surveillance. J Urol 2008; 179: 1804-9. (PMID: 18353398)
26. Tomlins, S. A., Rhodes, D. R., Perner, S., Dhanasekaran, S. M., Mehra, R., Sun, XW, Varambally S, Cao X, Tchinda J, Kuefer R, Lee C, Montie JE, Shah RB, Pienta KJ, Rubin MA, Chinnaiyan AM. Recurrent fusion of TMPRSS2 and ETS transcription factor genes in prostate cancer. Science, 310: 2005.
27. Tomlins, S. A., Mehra, R., Rhodes, DR, Smith LR, Roulston D, Helgeson BE, Cao X, Wei JT, Rubin MA, Shah RB, Chinnaiyan AM. TMPRSS2:ETV4 gene fusions define a third molecular subtype of prostate cancer. Cancer Research, 66: 3396, 2006.
28. Perner, S., Demichelis, F., Beroukhim, R., Schmidt FH, Mosquera JM, Setlur S, Tchinda J, Tomlins SA, Hofer MD, Pienta KG, Kuefer R, Vessella R, Sun XW, Meyerson M, Lee C, Sellers WR, Chinnaiyan AM, Rubin MA. TMPRRS2:ERG fusion-associated deletions provide insight into the heterogeneity of prostate cancer. Cancer Research, 66: 8337, 2006.
29. Lapointe, J., Kim, Y. H., Miller AM, Li C, Kaygusuz G, de Rijn MV, Huntsman DG, Brooks JD, Pollack JR, A variant TMPRSS2 isoform and ERG fusion product in prostate cancer with implications for molecular diagnosis. Modern Pathology, 20: 467, 2007.
30. Tomlins, S. A., Laxman, B., Dhanasekaran, S. M., Helgeson BE, Cao X, Morris DS, Menon A, Jing X, Cao Q, Han B, Yu J, Wang L, Montie JE, Rubin MA, Pienta KJ, Roulston D, Shah RB, Varambally S, Mehra R, Chinnaiyan AM. Distict classes of chromosomal rearrangements create oncogenic ETS gene fusions in prostate cancer. Nature, 448: 595, 2007.
31. Tu, J. J., Rohan, S., Kao, J., Kitabayashi N, Mathew S, Chen YT. Gene fusions between TMPRSS and ETS family genes in prostate cancer: frequency and transcript variant analysis by RT-PCR and FISH on paraffin-embedded tissue. Modern Pathology, 20: 921, 2007.
32. Perner, S., Demichelis, F., Beroukhim, R., Schmidt FH, Mosquera JM, Setlur S, Tchinda J, Tomlins SA, Hofer MD, Pienta KG, Kuefer R, Vessella R, Sun XW, Meyerson M, Lee C, Sellers WR, Chinnaiyan AM, Rubin MA.. TMPRSS2:ERG fusion-associated deletions provide insight into the heterogeneity of prostate cancer. Cancer Research, 66: 8337, 2006.
33. Demichelis, F., Fall, K., Perner, S., Adren, O., Schmidt, F., [Setlur SR](http://www.ncbi.nlm.nih.gov/sites/entrez?Db=pubmed&Cmd=Search&Term="Setlur SR"%5BAuthor%5D&itool=EntrezSystem2.PEntrez.Pubmed.Pubmed_ResultsPanel.Pubmed_DiscoveryPanel.Pubmed_RVAbstractPlus), [Hoshida Y](http://www.ncbi.nlm.nih.gov/sites/entrez?Db=pubmed&Cmd=Search&Term="Hoshida Y"%5BAuthor%5D&itool=EntrezSystem2.PEntrez.Pubmed.Pubmed_ResultsPanel.Pubmed_DiscoveryPanel.Pubmed_RVAbstractPlus), [Mosquera JM](http://www.ncbi.nlm.nih.gov/sites/entrez?Db=pubmed&Cmd=Search&Term="Mosquera JM"%5BAuthor%5D&itool=EntrezSystem2.PEntrez.Pubmed.Pubmed_ResultsPanel.Pubmed_DiscoveryPanel.Pubmed_RVAbstractPlus), [Pawitan Y](http://www.ncbi.nlm.nih.gov/sites/entrez?Db=pubmed&Cmd=Search&Term="Pawitan Y"%5BAuthor%5D&itool=EntrezSystem2.PEntrez.Pubmed.Pubmed_ResultsPanel.Pubmed_DiscoveryPanel.Pubmed_RVAbstractPlus), [Lee C](http://www.ncbi.nlm.nih.gov/sites/entrez?Db=pubmed&Cmd=Search&Term="Lee C"%5BAuthor%5D&itool=EntrezSystem2.PEntrez.Pubmed.Pubmed_ResultsPanel.Pubmed_DiscoveryPanel.Pubmed_RVAbstractPlus), [Adami HO](http://www.ncbi.nlm.nih.gov/sites/entrez?Db=pubmed&Cmd=Search&Term="Adami HO"%5BAuthor%5D&itool=EntrezSystem2.PEntrez.Pubmed.Pubmed_ResultsPanel.Pubmed_DiscoveryPanel.Pubmed_RVAbstractPlus), [Mucci LA](http://www.ncbi.nlm.nih.gov/sites/entrez?Db=pubmed&Cmd=Search&Term="Mucci LA"%5BAuthor%5D&itool=EntrezSystem2.PEntrez.Pubmed.Pubmed_ResultsPanel.Pubmed_DiscoveryPanel.Pubmed_RVAbstractPlus), [Kantoff PW](http://www.ncbi.nlm.nih.gov/sites/entrez?Db=pubmed&Cmd=Search&Term="Kantoff PW"%5BAuthor%5D&itool=EntrezSystem2.PEntrez.Pubmed.Pubmed_ResultsPanel.Pubmed_DiscoveryPanel.Pubmed_RVAbstractPlus), [Andersson SO](http://www.ncbi.nlm.nih.gov/sites/entrez?Db=pubmed&Cmd=Search&Term="Andersson SO"%5BAuthor%5D&itool=EntrezSystem2.PEntrez.Pubmed.Pubmed_ResultsPanel.Pubmed_DiscoveryPanel.Pubmed_RVAbstractPlus), [Chinnaiyan AM](http://www.ncbi.nlm.nih.gov/sites/entrez?Db=pubmed&Cmd=Search&Term="Chinnaiyan AM"%5BAuthor%5D&itool=EntrezSystem2.PEntrez.Pubmed.Pubmed_ResultsPanel.Pubmed_DiscoveryPanel.Pubmed_RVAbstractPlus), [Johansson JE](http://www.ncbi.nlm.nih.gov/sites/entrez?Db=pubmed&Cmd=Search&Term="Johansson JE"%5BAuthor%5D&itool=EntrezSystem2.PEntrez.Pubmed.Pubmed_ResultsPanel.Pubmed_DiscoveryPanel.Pubmed_RVAbstractPlus), [Rubin MA](http://www.ncbi.nlm.nih.gov/sites/entrez?Db=pubmed&Cmd=Search&Term="Rubin MA"%5BAuthor%5D&itool=EntrezSystem2.PEntrez.Pubmed.Pubmed_ResultsPanel.Pubmed_DiscoveryPanel.Pubmed_RVAbstractPlus). TMPRSS2:ERG gene fusion associated with lethal prostate cancer in a watchful waiting cohort. Oncogene 2007, 26931): 4596-4599.
34. Attard, G., Clark, J., Ambroisine, L., Fisher, **G**.; Kovacs, **G**.; Flohr, P.; Berney, D.; Foster, C. S.; Fletcher, A.; Gerald, W. L.; Moller, H.; Reuter, V.; De Bono, J. S.; Scardino, P.; Cuzick, J.; Cooper, C. S.. Duplication of the fusion TMPRSS2 to ERG sequence identifies fatal human prostate cancer. Oncogene, 2008; 27(3): 253-263.
35. Laxman, B., Tomlins, S. A., Mehra, R.,Morris DS, Wang L, Helgeson BE, Shah R, Rubinb MA, Wei JT, Chinnaiyan AM. Noninvasive detection of TMPRSS2:ERG fusion transcripts in urine of men with prostate cancer. Neoplasia, 8: 885-888, 2006.
36. Hessels, D., Smit, F. P., Verhaegh, G. W., Witjes, J. A., Cornel, E. B., and Schalken, J. A.: Detection of TMPRSS-2-ERG fusion transcripts and prostate cancer antigen in urinary sediments may improve diagnosis of prostate cancer. Clinical Cancer Research, 13: 5103, 2007.
37. Groskopf, J., Siddiqui, J., Sefton-Miller, Fradet, Y., Schalken, J., Rittenhouse, H., Chinnaiyan, A. Feasibility and clinical utility of a TMPRSS2:ERG gene fusion urine test. Presented at 2009 ASCO GU Symposium, Orlando, FL.
38. Cairns P, Esteller M, Herman JG, Schoenberg M, Jeronimo C, Sanchez-Cespedes M, Chow NH, Grasso M, Wu L, Westra WB, Sidransky D. Molecular detection of prostate cancer in urine by GSTP1 hypermethylation. Clin Cancer Res 2001;7:2727– 2730.
39. Jeronimo C, Usadel H, Henrique R, Silva C, Oliveira J, Lopes C, Sidransky D. Quantitative GSTP1 hypermethylation in bodily fluids of patients with prostate cancer. Urology 2002;60:1131–1135.
40. Gonzalgo ML, Pavlovich CP, Lee SM, Nelson WG. Prostate cancer detection by GSTP1 methylation analysis of postbiopsy urine specimens. Clin Can Res 2003;9:2673–7.
41. Hoque MO, Topaloglu O, Begum S, Henrique R, Rosenbaum E, Criekinge MV, Westra WH, Sidransky D. Quantitative methylation-specific polymerase chain reaction gene patterns in urine sediment distinguish prostate cancer patients from control subjects. J Clin Oncol 2005; 23: 6569-6575.
42. Rouprêt M, Hupertan V, Yates DR, Catto JWF, Rehman I, Meuth M, Ricci S, Lacave R, Cancel-Tassin G, de la Taille A, Rozet F, Cathelineau X, Vallancien G, Hamdy FC, Cussenot O. Molecular detection of localized prostate cancer using quantitative methylation-specific PCR on urinary cells obtained following prostate massage. Clin Cancer Res 2007; 13: 1720-1725.
43. Vener T, Derecho C, Baden J, Wang H, Rajpurohit Y, Skelton Mehrotra JJ, Varde S, Chowdary D, Stallings W, Leibovich B, Robin H, Pelzer A, Schäfer G, Auprich M, Mannweiler S, Amersdorfer P, Mazumder.A. Development of a multiplexed urine assay for prostate cancer diagnosis. Clin Chem 2008; 54: 874-882. First published as 10.1373/clinchem.2007.094912.
44. [Parekh DJ, Ankerst DP, Higgins BA, Hernandez J, Canby-Hagino E, Brand T, Troyer DA, Leach RJ, Thompson IM.](http://www.ncbi.nlm.nih.gov/sites/entrez?Db=pubmed&Cmd=ShowDetailView&TermToSearch=17169636&ordinalpos=4&itool=EntrezSystem2.PEntrez.Pubmed.Pubmed_ResultsPanel.Pubmed_RVDocSum) External validation of the Prostate Cancer Prevention Trial risk calculator in a screened population. Urology. 2006;68:1152-5.
45. Hernandez DJ, Han M, Humphreys EB, Mangold LA. External validation of the prostate cancer risk calculator. J Urology 2006;177:622.
46. Han M, Humphreys EB, Hernandez DJ, Partin AW. Comparison between the Prostate Cancer Risk Calculator and PSA. J Urology 2006;177:622.
47. Reed A, Ankerst DP, Pollock BH, Thompson IM, Parekh DJ. Current age- and race-adjusted PSA threshold values delay diagnosis of high grade prostate cancer. J Urology, 2007;178:1929-32.
48. [Thompson IM, Chi C, Ankerst DP, Goodman PJ, Tangen CM, Lippman SM, Lucia MS, Parnes HL, Coltman CA Jr.](http://www.ncbi.nlm.nih.gov/sites/entrez?Db=pubmed&Cmd=ShowDetailView&TermToSearch=16912265&ordinalpos=4&itool=EntrezSystem2.PEntrez.Pubmed.Pubmed_ResultsPanel.Pubmed_RVDocSum) Effect of finasteride on the sensitivity of PSA for detecting prostate cancer. J Natl Cancer Inst. 2006;98:1128-33.
49. [Thompson IM, Pauler Ankerst D, Chi C, Goodman PJ, Tangen CM, Lippman SM, Lucia MS, Parnes HL, Coltman CA Jr.](http://www.ncbi.nlm.nih.gov/sites/entrez?Db=pubmed&Cmd=ShowDetailView&TermToSearch=17634486&ordinalpos=1&itool=EntrezSystem2.PEntrez.Pubmed.Pubmed_ResultsPanel.Pubmed_RVDocSum) Prediction of prostate cancer for patients receiving finasteride: results from the prostate cancer prevention trial. J Clin Oncol. 2007;25:3076-81.
50. Handel LN, Agarwal S, Schiff SF, Kelty PJ, Cohen SI. Can effect of finasteride on prostate-specific antigen be used to decrease repeat prostate biopsy? Urology 2006;68:1220-23.
51. Khan MA, Mangold LA, Epstein JI, Boitnott JK, Walsh PC, Partin AW. Impact of surgical delay on long-term cancer control for clinically localized prostate cancer. J Urology 2004;172:1835-9.
52. Freedland SJ, Kane CJ, Amling CL, Aronson WJ, Presti J, Terris M. Delay of radical prostatectomy and risk of biochemical progression in men with low risk prostate cancer. J Urology 2006;175:1298-302.
53. The Finasteride Study Group. Finasteride (MK-906) in the treatment of benign prostatic hyperplasia. The Prostate 22:291-99, 1993.
54. [Thompson IM, Tangen CM, Goodman PJ, Lucia MS, Parnes HL, Lippman SM, Coltman CA Jr.](http://www.ncbi.nlm.nih.gov/pubmed/17437804?ordinalpos=9&itool=EntrezSystem2.PEntrez.Pubmed.Pubmed_ResultsPanel.Pubmed_RVDocSum) Finasteride improves the sensitivity of digital rectal examination for prostate cancer detection. J Urol. 2007;177:1749-52.
55. [Lucia MS, Epstein JI, Goodman PJ, Darke AK, Reuter VE, Civantos F, Tangen CM, Parnes HL, Lippman SM, La Rosa FG, Kattan MW, Crawford ED, Ford LG, Coltman CA Jr, Thompson IM.](http://www.ncbi.nlm.nih.gov/pubmed/17848673?ordinalpos=3&itool=EntrezSystem2.PEntrez.Pubmed.Pubmed_ResultsPanel.Pubmed_RVDocSum) Finasteride and high-grade prostate cancer in the Prostate Cancer Prevention Trial. J Natl Cancer Inst. 2007;99:1375-83.
56. [Moinpour CM, Darke AK, Donaldson GW, Thompson IM Jr, Langley C, Ankerst DP, Patrick DL, Ware JE Jr, Ganz PA, Shumaker SA, Lippman SM, Coltman CA Jr.](http://www.ncbi.nlm.nih.gov/pubmed/17596576?ordinalpos=2&itool=EntrezSystem2.PEntrez.Pubmed.Pubmed_ResultsPanel.Pubmed_RVDocSum) Longitudinal analysis of sexual function reported by men in the Prostate Cancer Prevention Trial. J Natl Cancer Inst. 2007;99:1025-35.
57. [Thompson IM, Lucia MS, Redman MW, Darke A, La Rosa FG, Parnes HL, Lippman SM, Coltman CA.](http://www.ncbi.nlm.nih.gov/pubmed/17499284?ordinalpos=8&itool=EntrezSystem2.PEntrez.Pubmed.Pubmed_ResultsPanel.Pubmed_RVDocSum) Finasteride decreases the risk of prostatic intraepithelial neoplasia. J Urol. 2007;178:107-9.
58. Redman MW, Tangen CM, Goodman PJ, Lucia MS, Coltman CA, Thompson IM. Finasteride does not increase the risk of high-grade prostate cancer: A bias-adjusted modeling approach. Cancer Prevention Research 2008;1:174-81. (PMC – In Process)
59. Lucia MS, Darke AK, Goodman PJ, LaRosa F, Parnes HL, Ford LG, Coltman MA, Thompson IM. Pathologic characteristics of cancers detected in the Prostate Cancer Prevention Trial: Implications for prostate cancer detection and chemoprevention. Cancer Prevention Research 2008;1:167-73. (PMC Journal – In process)
60. [Baillargeon J, Platz EA, Rose DP, Pollock BH, Ankerst DP, Haffner S, Higgins B, Lokshin A, Troyer D, Hernandez J, Lynch S, Leach RJ, Thompson IM.](http://www.ncbi.nlm.nih.gov/sites/entrez?Db=pubmed&Cmd=ShowDetailView&TermToSearch=16835332&ordinalpos=55&itool=EntrezSystem2.PEntrez.Pubmed.Pubmed_ResultsPanel.Pubmed_RVDocSum) Obesity, adipokines, and prostate cancer in a prospective population-based study. Cancer Epidemiol Biomarkers Prev. 2006;15:1331-5.
61. [Baillargeon J, Pollock BH, Kristal AR, Bradshaw P, Hernandez J, Basler J, Higgins B, Lynch S, Rozanski T, Troyer D, Thompson I.](http://www.ncbi.nlm.nih.gov/sites/entrez?Db=pubmed&Cmd=ShowDetailView&TermToSearch=15668913&ordinalpos=110&itool=EntrezSystem2.PEntrez.Pubmed.Pubmed_ResultsPanel.Pubmed_RVDocSum) The association of body mass index and prostate-specific antigen in a population-based study. Cancer. 2005;103:1092-5.
62. [Semmes OJ, Feng Z, Adam BL, Banez LL, Bigbee WL, Campos D, Cazares LH, Chan DW, Grizzle WE, Izbicka E, Kagan J, Malik G, McLerran D, Moul JW, Partin A, Prasanna P, Rosenzweig J, Sokoll LJ, Srivastava S, Srivastava S, Thompson I, Welsh MJ, White N, Winget M, Yasui Y, Zhang Z, Zhu L.](http://www.ncbi.nlm.nih.gov/sites/entrez?Db=pubmed&Cmd=ShowDetailView&TermToSearch=15613711&ordinalpos=116&itool=EntrezSystem2.PEntrez.Pubmed.Pubmed_ResultsPanel.Pubmed_RVDocSum) Evaluation of serum protein profiling by surface-enhanced laser desorption/ionization time-of-flight mass spectrometry for the detection of prostate cancer: I. Assessment of platform reproducibility. Clin Chem. 2005;5:102-12.
63. [Grizzle WE, Semmes OJ, Basler J, Izbicka E, Feng Z, Kagan J, Adam BL, Troyer D, Srivastava S, Thornquist M, Zhang Z, Thompson IM.](http://www.ncbi.nlm.nih.gov/sites/entrez?Db=pubmed&Cmd=ShowDetailView&TermToSearch=15283893&ordinalpos=130&itool=EntrezSystem2.PEntrez.Pubmed.Pubmed_ResultsPanel.Pubmed_RVDocSum) The early detection research network surface-enhanced laser desorption and ionization prostate cancer detection study: A study in biomarker validation in genitourinary oncology. Urol Oncol. 2004;22:337-43.
64. [Grizzle WE, Adam BL, Bigbee WL, Conrads TP, Carroll C, Feng Z, Izbicka E, Jendoubi M, Johnsey D, Kagan J, Leach RJ, McCarthy DB, Semmes OJ, Srivastava S, Srivastava S, Thompson IM, Thornquist MD, Verma M, Zhang Z, Zou Z.](http://www.ncbi.nlm.nih.gov/sites/entrez?Db=pubmed&Cmd=ShowDetailView&TermToSearch=15258333&ordinalpos=133&itool=EntrezSystem2.PEntrez.Pubmed.Pubmed_ResultsPanel.Pubmed_RVDocSum) Serum protein expression profiling for cancer detection: validation of a SELDI-based approach for prostate cancer. Dis Markers. 2003-2004;19:185-95.
65. [Balic I, Graham ST, Troyer DA, Higgins BA, Pollock BH, Johnson-Pais TL, Thompson IM, Leach RJ.](http://www.ncbi.nlm.nih.gov/sites/entrez?Db=pubmed&Cmd=ShowDetailView&TermToSearch=12394768&ordinalpos=206&itool=EntrezSystem2.PEntrez.Pubmed.Pubmed_ResultsPanel.Pubmed_RVDocSum) Androgen receptor length polymorphism associated with prostate cancer risk in Hispanic men. J Urol. 2002;168:2245-8.
66. Lachenbruch PA. On the sample size for studies based upon McNemar’s test. Statistics in Medicine 1992; 11: 1521-25.
67. DeLong ER, DeLong DM, Clarke-Pearson DL. Comparing the areas under two or more correlated receiver operating characteristic curves: a nonparametric approach. Biometrics 1988;44:837-45.
68. McConnell JD, Roehrborn CG, Bautista OM, Andriole GL Jr, Dixon CM, Kusek JW, Lepor H, McVary KT, Nyberg LM Jr, Clarke HS, Crawford ED, Diokno A, Foley JP, Foster HE, Jacobs SC, Kaplan SA, Kreder KJ, Lieber MM, Lucia MS, Miller GJ, Menon M, Milam DF, Ramsdell JW, Schenkman NS, Slawin KM, Smith JA; Medical Therapy of Prostatic Symptoms (MTOPS) Research Group.  The long-term effect of doxazosin, finasteride, and combination therapy on the clinical progression of benign prostatic hyperplasia.  N Engl J Med. 2003 Dec 18;349(25):2387-98.

Appendix I
